# Synthesis and in-depth structure determination of a novel metastable high-pressure CrTe_3_ phase

**DOI:** 10.1107/S1600576724002711

**Published:** 2024-05-24

**Authors:** Lennart Voss, Nico Alexander Gaida, Anna-Lena Hansen, Martin Etter, Niklas Wolff, Viola Duppel, Andriy Lotnyk, Wolfgang Bensch, Hubert Ebert, Sergey Mankovsky, Svitlana Polesya, Shrikant Bhat, Robert Farla, Masashi Hasegawa, Takuya Sasaki, Ken Niwa, Lorenz Kienle

**Affiliations:** a Department of Materials Science, Synthesis and Real Structure, Christian-Albrechts-University Kiel, Kaiserstrasse 2, Kiel, 24143, Germany; b Department of Materials Physics, Nagoya University, Furo-cho, Chikusa-ku, Nagoya, 464-8601, Japan; c Institute for Applied Materials – Energy Storage Systems (IAM-ESS), Karlsruhe Institute of Technology (KIT), Hermann-von-Helmholtz-Platz 1, Eggenstein-Leopoldshafen, 76344, Germany; d Deutsche Elektronen-Synchrotron DESY, Notkestrasse 85, Hamburg, 22607, Germany; e Kiel Nano, Surface and Interface Science KiNSIS, Kiel University, Christian-Albrechts-Platz 4, Kiel, 24118, Germany; f Max Planck Institute for Solid State Research, Heisenbergstrasse 1, Stuttgart, 70569, Germany; g Leibniz Institute for Surface Modification (IOM), Permoserstrasse 15, Leipzig, 04318, Germany; h Institute of Inorganic Chemistry, Christian-Albrechts-University Kiel, Max-Eyth Strasse 2, Kiel, 24118, Germany; i Department Chemie, Physikalische Chemie, Universität München, Butenandtstrasse 5-13, München, D-81377, Germany; SLAC National Accelerator Laboratory, Menlo Park, USA

**Keywords:** chromium telluride, synchrotron diffraction, structure determination, density functional theory, DFT

## Abstract

This study reports the synthesis and crystal structure determination of a novel CrTe_3_ phase using various experimental and theoretical methods. The average stoichiometry and local phase separation of this quenched high-pressure phase were characterized by *ex situ* synchrotron powder X-ray diffraction and total scattering. Several structural models were obtained using simulated annealing, but all suffered from an imperfect Rietveld refinement, especially at higher diffraction angles. Finally, a novel stoichiometrically correct crystal structure model was proposed on the basis of electron diffraction data and refined against powder diffraction data using the Rietveld method. Scanning electron microscopy–energy-dispersive X-ray spectrometry (EDX) measurements verified the targeted 1:3 (Cr:Te) average stoichiometry for the starting compound and for the quenched high-pressure phase within experimental errors. Scanning transmission electron microscopy (STEM)–EDX was used to examine minute variations of the Cr-to-Te ratio at the nanoscale. Precession electron diffraction (PED) experiments were applied for the nanoscale structure analysis of the quenched high-pressure phase. The proposed monoclinic model from PED experiments provided an improved fit to the X-ray patterns, especially after introducing atomic anisotropic displacement parameters and partial occupancy of Cr atoms. Atomic resolution STEM and simulations were conducted to identify variations in the Cr-atom site-occupancy factor. No significant variations were observed experimentally for several zone axes. The magnetic properties of the novel CrTe_3_ phase were investigated through temperature- and field-dependent magnetization measurements. In order to understand these properties, auxiliary theoretical investigations have been performed by first-principles electronic structure calculations and Monte Carlo simulations. The obtained results allow the observed magnetization behavior to be interpreted as the consequence of competition between the applied magnetic field and the Cr–Cr exchange interactions, leading to a decrease of the magnetization towards *T* = 0 K typical for antiferromagnetic systems, as well as a field-induced enhanced magnetization around the critical temperature due to the high magnetic susceptibility in this region.

## Abstract 2

High-pressure/high-temperature experiments using monoclinic CrTe_3_ as a starting phase result in a novel structural polymorph identified as CrTe_3_ (*P*2/*m*) using synchrotron X-ray diffraction and transmission electron microscopy. The magnetic properties of the novel phase were investigated through temperature- and field-dependent magnetization measurements and correlated with auxiliary theoretical investigations by first-principles electronic structure calculations and Monte Carlo simulations.

## 1. Introduction

Cr tellurides are a family of compounds that exhibit diverse structural and magnetic properties. Among them, several phases adopt the NiAs-like aristotype structure: Cr_1−*x*_Te, Cr_7_Te_8_, Cr_3_Te_4_, Cr_2_Te_3_ and dimorphic Cr_5_Te_8_ (Chevreton *et al.*, 1963 ▸; Ipser *et al.*, 1983 ▸; Bensch *et al.*, 1997 ▸; Chattopadhyay, 1994 ▸). These phases are characterized by their ferromagnetic behavior with relatively high Curie temperatures *T*_C_ between −103 and 67°C (see *e.g.* Akram & Nazar, 1983 ▸; Lukoschus *et al.*, 2004 ▸; Huang *et al.*, 2008 ▸; Dijkstra *et al.*, 1989 ▸). On the other hand, the two Te-rich phases CrTe_2_ (Zhang *et al.*, 1990 ▸) and CrTe_3_ (Klepp & Ipser, 1979 ▸, 1982 ▸) crystallize in layered structures that differ from the NiAs aristotype.

While Cr tellurides have not been the focus of research for a long time, a renaissance is now being observed because some of the compounds exhibit unusual properties. For epitaxial thin CrTe films, an anomalous Hall effect was discovered, which is in accordance with a topological Hall effect in chiral magnets with a skyrmion phase (Zhao *et al.*, 2018 ▸). The magnitude of *T*_C_ can be systematically tuned in ferromagnetic Cr_5+_ *_x_* Te_8_ by adjusting the Cr content, reaching a value of 40°C for *x* = 1 (Zhang, He *et al.*, 2020 ▸). Phase engineering of Cr_5_Te_8_ led to the detection of a colossal anomalous Hall effect (Tang *et al.*, 2022 ▸). Starting with the layered compound CrTe_2_ several lateral and vertical magnetic heterojunctions could be realized via self-intercalation, such as a lateral Cr_2_Te_3_–Cr_5_Te_8_ heterojunction exhibiting unusual magneto-optical behavior, which is important for spintronic devices (Niu *et al.*, 2023 ▸). An anisotropic magnetocaloric effect Δ*S*_m_ was reported for the trigonal polymorph of Cr_5_Te_8_ with negative values in the *a*–*b* plane and a positive value along the *c* axis (Liu *et al.*, 2019 ▸). Theoretical calculations predict a *T*_C_ value of 187°C for monolayer Cr_3_Te_4_ (Zhang *et al.*, 2019 ▸), and experimentally magnetic skyrmion behavior was found for ultra-thin Cr_3_Te_4_ (Li, Deng *et al.*, 2022 ▸) as well as a *T*_C_ of 71°C for monolayer Cr_3_Te_4_ (Chua *et al.*, 2021 ▸).

A thickness-dependent *T*_C_ was reported for ultra-thin Cr_2_Te_3_, which reached 7°C for a film thickness of six unit cells (Wen *et al.*, 2020 ▸). An even higher *T*_C_ of 22°C was reported for epitaxial thin films of Cr_2_Te_3_, making this compound interesting for spintronic applications (Li *et al.*, 2019 ▸). In ferromagnetic quasi-2D Cr_1.2_Te_2_, which was prepared by a reaction between KCrTe_2_ and I_2_/aceto­nitrile, a topological Hall effect was observed over a large temperature range up to 47°C (Huang *et al.*, 2021 ▸). The new compound Cr_4_Te_5_, which can be regarded as a self-intercalated CrTe_2_, exhibits a 3D Heisenberg-like magnetic behavior with *T*_C_ = 45.5°C (Zhang, Zhang *et al.*, 2020 ▸).

These examples reveal the unusual properties of Cr-rich tellurides and suggest the potential for discovering more interesting physical phenomena through further research. Four decades ago, CrTe_3_ was first synthesized by a solid-state reaction and found to be a thermodynamically stable phase (Klepp & Ipser, 1979 ▸). However, this compound has received little attention since then. In 2002 it was demonstrated that this compound can be obtained as thin films at a temperature as low as 100°C (Kraschinski *et al.*, 2002 ▸). Theoretical investigations of the electronic situation showed the presence of a polymeric telluride network composed of Te^2−^, Te_2_^2−^ and Te_3_^2−^ anions (Canadell *et al.*, 1992 ▸). According to the calculations, this thermodynamically stable CrTe_3_ phase is a Mott–Hubbard semiconductor with a small band gap. The magnetic properties are governed by antiferromagnetic interactions within a Cr_4_ tetramer and ferromagnetic exchange between the tetramers (McGuire *et al.*, 2017 ▸). Temperature-dependent X-ray diffraction experiments showed an abrupt structural distortion at *T* ≃ −23°C, which remains constant to 173°C. The structural distortion is reflected in the occurrence of a substantial spontaneous magnetization at *T* < −33°C, indicating ferro- or ferrimagnetic exchange interactions (Hansen *et al.*, 2018 ▸). Epitaxially grown antiferromagnetic monolayers (MLs) of CrTe_3_ in combination with MLs of metallic magnetic CrTe_2_ were used for the fabrication of lateral metal–semiconductor heterojunctions (Yao *et al.*, 2022 ▸). Vacuum annealing of thin CrTe_3_ films led to the partial transformation into CrTe_2_, thus forming planar CrTe_3_–CrTe_2_ heterojunctions with atomically sharp interfaces, which may be used in spintronic devices (Li, Nie *et al.*, 2022 ▸).

Results of combined X-ray diffraction and magnetic measurements demonstrate that CrTe shows a negative thermal expansion behavior (7–67°C), Cr_3_Te_4_ exhibits zero thermal expansion properties (−93–47°C), and for trigonal Cr_5_Te_8_ a positive thermal expansion was found between −170 and 227°C (Li, Liu, Jiang *et al.*, 2022 ▸).

High pressure significantly modifies electronic structures and interatomic bonding as well as downsizing atomic distances, which often favors the discovery of unique materials that cannot be synthesized under atmospheric pressure. Hence, novel materials that are inaccessible at ambient conditions can be synthesized. The *T*_C_ value of 69°C at room temperature of nearly stoichiometric CrTe (Cr_48_Te_52_) significantly decreased by −53°C GPa^−1^ and at 5–7 GPa a value of −203°C was reached. On the basis of further characterizations, a pressure-induced magnetic phase transition was postulated to occur at ∼7 GPa (Ishizuka *et al.*, 2001 ▸). For Cr_2_Te_3_ the value for *T*_C_ is reduced with increasing pressure by −1.78°C kbar^−1^ (Yuzuri *et al.*, 1987 ▸). The spontaneous mag­netization of Cr_2_Te_3_ at −268.8°C decreases with increasing pressure, while for Cr_5_Te_8_ almost no effect was observed (Kanomata *et al.*, 1998 ▸). The reduction of *T*_C_ was also reported for Cr_3_Te_4_ and Cr_7_Te_8_ (Ozawa *et al.*, 1972 ▸; Ohta *et al.*, 1996 ▸).

For the three phases CrTe, Cr_3_Te_4_ and Cr_5_Te_8_ significant alterations of the structural and selected physical properties were observed under high pressure. The most Cr-rich phase CrTe undergoes a structural phase transition from the NiAs to the MnP type at ∼15 GPa, whereas an isostructural phase transition is observed for Cr_3_Te_4_ (∼12 GPa) and Cr_5_Te_8_ (∼11 GPa). Moreover, a semiconductor-to-metal transition was found for CrTe (∼24 GPa) and Cr_3_Te_4_, while Cr_5_Te_8_ underwent metal–semiconductor–metal transitions during increasing compression. Pressure-induced *n*–*p*-type conduction transitions were observed for CrTe and Cr_5_Te_8_, while Cr_3_Te_4_ exhibited *p*-type conduction in the whole pressure range (Li, Liu, Jin *et al.*, 2022 ▸).

Previous studies have explored the structural and physical properties of Cr tellurides under pressure, but the high-pressure high-temperature behavior of CrTe_3_ remains unknown. To fill this gap, we performed *in situ* X-ray diffraction experiments on CrTe_3_ at various pressures and temperatures. In this way a new polymorph of CrTe_3_ could be obtained, whose structure was solved using dedicated *ex situ* methods. Furthermore, the magnetic properties of the novel CrTe_3_ phase were investigated through temperature- and field-dependent magnetization measurements, and the electronic structure was calculated from the experimental structure data set of the new CrTe_3_ polymorph.

## 2. Experimental methods

### 2.1. Synthesis of the starting CrTe_3_ material

The starting material CrTe_3_ used for high-pressure studies was synthesized using a high-temperature approach described by Hansen *et al.* (2018 ▸). A stoichiometric mixture of Cr and Te was sealed in an evacuated quartz ampoule that was heated to 300°C for 24 h and then ramped up to 450°C. This temperature was held for 4 d, before slow cooling of the sample to room temperature.

### 2.2. *In situ* synchrotron diffraction under high pressure and temperature

*In situ* synchrotron X-ray diffraction experiments at high pressure (*P*) and temperature (*T*) were performed with a modified cubic large-volume press (mavo press, Max Voggenreiter GmbH) located at the beamline ID06-LVP, ESRF. The cross section of the octahedral high-*PT* cell is shown in Fig. S1 in the supporting information. The second-stage anvils were tungsten carbide cubes with a truncated edge length of 4 mm equipped with pyrophyllite gaskets. These anvils enclose the pressure medium consisting of a 5% Cr_2_O_3_-doped octahedral MgO cell with an edge length of 10 mm which contains the sample and a pressure/temperature marker (Pt and MgO) surrounded by a BN sleeve, along with a rhenium foil resistance furnace and ZrO_2_ insulating plugs. Along the beam direction, cylindrical SiBCN X-ray windows and ∼4 mm-wide boron rectangles were inserted into the octahedra and gaskets, respectively (Fig. S1). The high-*PT* cell was compressed at a rate of 0.04 GPa min^−1^ to a corresponding pressure of 10.5 GPa and then heated via the rhenium resistance furnace at a rate of ∼5°C min^−1^. After the observed phase transformation at ∼230°C, the temperature was held for ∼10 min, and then the reaction was immediately quenched. Pressures and temperatures were calibrated *in situ* from X-ray diffraction patterns using Pt and h-BN (h denotes hexagonal) equations of state (cross-calibration between Pt and h-BN). X-ray diffraction (XRD) patterns were continuously collected at a constant wavelength (λ = 0.2296 Å) select­ed by a Si(111) double-crystal monochromator from the emission of a U18 insertion device at a ∼6 mm magnetic gap. Data acquisition in a 2θ range of 2–10° was performed via a Detection Technology X-Scan series 1 linear pixelated detec­tor. LaB_6_-SRM660a (NIST) was employed for the calibration of the sample-to-detector distance and the detector offset. The data were integrated and analyzed using the *FIT2D* (Ham­mersley, 2016 ▸; https://www.esrf.fr/computing/scientific/FIT2D/) and *PDindexer* (https://pandas.pydata.org/docs/reference/api/pandas.Index.html) software. The novel CrTe_3_ phase obtained in the high-*PT* experiments was recovered and was used for further characterization.

### 2.3. Synthesis of the discovered CrTe_3_ compound

The high pressures were applied by the transformation of uniaxial forces of hydraulic presses into quasi-hydro­static pressure using a DIA-type multi-anvil press at the Department of Materials Physics, Nagoya University (Japan), and a 6-rams large-volume press (LVP) (mavo press LPQ6 1500/100, Max Voggenreiter GmbH) at the P61B beamline (DESY, Hamburg, Germany). In both apparatuses, cemented tungsten carbide (WC) second-stage anvils were used with truncated edge lengths of 6 and 15 mm, respectively. These anvils com­pressed a cubic pressure medium consisting of pyrophyllite with an edge length of 8 mm (and 20 mm for the 6-rams LVP) equipped with a cylindrical carbon resistive heater inside. Pressures were calibrated *ex situ* without external heating using pressure-dependent phase transitions of bis­muth and barium, while temperatures were monitored *in situ* with an R-type (Pt 13%Rh–Pt) thermocouple. The cell assembly was performed in a glovebox to reduce the oxygen contamination. For syntheses, the pressure was incrementally increased over ∼40 min to the final values of 6 GPa without external heating. Next, the temperature was applied to the target value of 250°C with a heating rate of ∼100°C min^−1^ and then held for 10 min. Immediately after heating, quenching was initiated, followed by the pressure release. The novel CrTe_3_ phase was formed in the additional *ex situ* syntheses.

### 2.4. *Ex situ* synchrotron scattering experiments

*Ex situ* synchrotron powder X-ray diffraction experiments at ambient conditions were performed at the powder diffraction and total scattering beamline P02.1 (DESY, Hamburg, Germany) by loading the high-pressure-synthesized CrTe_3_ powder pellet (recovered ESRF phase) into a Kapton capillary tube. Diffraction data were collected with a PerkinElmer XRD 1621 CN3–EHS area detector for an integration time of 180 s at a fixed wavelength of λ = 0.20722 Å while the capillary was spun for better statistics. LaB_6_-SRM660b (NIST) was employed for the calibration of the sample-to-detector distance and the detector offset. The data were integrated using the *DAWN Science* software (Basham *et al.*, 2015 ▸; Filik *et al.*, 2017 ▸) to a 1D pattern. Subsequent analysis of the diffrac­tion data was done using the *TOPAS6.0* software (Coelho, 2018 ▸; Bruker, 2017 ▸).

At the same beamline, *ex situ* total scattering experiments were performed as the basis for the pair distribution function (PDF) analysis using the same setup at a shorter sample-to-detector distance of 220 mm and a wavelength of λ = 0.20723 Å. LaB_6_-SRM660b (NIST) was measured under the same conditions for calibration of the sample-to-detector distance, the detector offset and the instrument contribution to the PDF (*Q*_damp_ = 0.38 Å^−1^). The data were integrated using the *DAWN Science* software. An empty capillary was measured and subtracted from the data before Fourier transformation. The calculation of the corresponding PDF was performed using *PDFgetX3* using a *Q*_max_ of 27.11 Å^−1^ (Juhás *et al.*, 2013 ▸). Real-space Rietveld refinement and modeling of PDFs were performed using *PDFgui* (Billinge & Farrow, 2013 ▸).

XRD measurements were also conducted at BL2S1, Aichi Synchrotron Radiation Center, Aichi, Japan (Watanabe *et al.*, 2017 ▸). The sample attached to the polyimide capillary was irradiated by incident X-rays with a wavelength of 0.75 Å and a beam size of 75 µm. The sample was rotated to get smooth diffraction lines during the X-ray irradiation, and the diffracted X-rays were detected with a 2D detector with an exposure time of 100 s.

### 2.5. Structure solution and refinement

The rather high symmetry space group *Pnn*2 was assumed for a first structural solution by the simulated annealing approach (Coelho, 2000 ▸). For the Rietveld refinements the fundamental parameter method was used as implemented in the *TOPAS* software (Rietveld, 1969 ▸; Rebuffi *et al.*, 2017 ▸), first with isotropic and later with anisotropic displacement parameters. For the search for possible crystallographic subgroups the *ISODISTORT* software was used (Campbell *et al.*, 2006 ▸). To check if the symmetry was determined correctly *PLATON* (Campbell *et al.*, 2006 ▸) was applied. Further, precession electron diffraction (PED) (see below) points to the alternative monoclinic space group *P*2/*m*.

The crystallographic parameters and the Rietveld refinement parameters of the novel CrTe_3_ crystal structure are listed in Tables 1 ▸ and 2 ▸, respectively. All parameters are physically sound, although it is very likely that the modeled anisotropic displacement parameters especially for the low-occupancy Cr position might be under- or overestimated.

### 2.6. Electron microscopy techniques

The averaged stoichiometry of the pristine CrTe_3_ phase and the quenched novel high-pressure phase of CrTe_3_ powders was analyzed by energy-dispersive X-ray spectroscopy (EDX) on a Vega TS 3150 MM scanning electron microscope equipped with an Oxford X-Max^N^ 20 detector [silicon drift detector (SDD) with an active area of 20 mm^2^]. Thereby, the determination of the elemental composition of the pristine CrTe_3_ phase also served as a standard for calibration and comparison with the quenched novel phase. The quenched crystal powders were further investigated for local variation of the average stoichiometry by recording large-area elemental maps across multiple grains by scanning transmission electron microscopy (STEM)–EDX on an FEI Titan3 G2 60–300 microscope (operated at 300 kV) equipped with a 4-SDD Super-X EDX system (30 mm^2^ each, EDX solid angle ∼0.7 sr). For transmission electron microscopy (TEM) analyses, the powder was embedded in ep­oxy resin which was sliced into a lamella with a thickness of <100 nm by the focused ion-beam method (FIB). Aberration-corrected STEM images showing the atomic structural motif of the Te sublattice were recorded with atomic resolution using *Z*-contrast imaging with a high-angle annular dark-field (HAADF-STEM) detector using annular ranges of 80–200 mrad. In addition, model-based simulations of the atomic *Z*-contrast images were conducted using the *Dr Probe* software package (Barthel, 2018 ▸) and compared with the experimental micrographs.

Nanoscale structure analysis of multiple grains from the quenched high-pressure phase was conducted using electron diffraction experiments via TEM. In particular, as well as the arrangement of the reflections, their intensity distribution is highly relevant for reliable structure identification of the material under investigation (Jones *et al.*, 1977 ▸; Vainshtein, 2013 ▸). Such detail was provided by PED experiments on a Philips CM30 ST (300 kV, LaB_6_ cathode, Cs = 1.15 mm) microscope equipped with a Spinning Star device (Nanomegas), which effectively limits the influence of dynamic electron scattering, resulting in more kinematic reflection intensity distributions and an increase in the spatial resolution up to higher-order Laue zones (Oleynikov *et al.*, 2007 ▸). This feature is achieved by recording diffraction patterns with an off-axis tilt of the primary beam and a 360° precession motion and subsequently averaging the collected diffracted intensity over all patterns. The powders were prepared by a conventional drop-casting method after immersion in *n*-butanol on a Lacey-carbon/copper TEM grid. The experimental PED patterns are compared with simulated PED patterns based on the refined preliminary structural solutions of CrTe_2_ (*Pnn*2), CrTe_4_ (*P*2_1_ */m*) and the proposed CrTe_3_ (*P*2*/m*) using the *JEMS-EMS* Java Version V4 and *Diamond* (V.4.6.8) software packages (Stadelmann, 2003 ▸) for structure visualization and modification.

### 2.7. Superconducting quantum interference device magnetometry

Magnetic characterization of well sintered cylindrical bulk CrTe_3_ samples with dimensions of ∼1.5 × 1.5 mm was performed by magnetometry (Quantum Design MPMS3) using a superconducting quantum interference device (SQUID) setup in the temperature range 300 to 2 K and with external applied fields of 30 mT. Samples were carefully prepared according toliterature suggestions for measurements of small samples with weak magnetic signals, *i.e.* glued into straws (both diamagnetic) and then transferred into the SQUID (Garcia *et al.*, 2009 ▸; Buchner *et al.*, 2018 ▸).

### 2.8. Theoretical calculations

The electronic and magnetic properties of the novel CrTe_3_ phase have been investigated by means of first-principles density functional theory (DFT) calculations using the spin-polarized relativistic Korringa–Kohn–Rostoker method (*SPR-KKR*, https://www.ebert.cup.uni-muenchen.de/en/software-en/13-sprkkr; Ebert *et al.*, 2011 ▸). The exchange-correlation potential was calculated within the local spin-density approximation using the parametrization as suggested by Vosko *et al.* (1980 ▸). The chemical disorder within the Cr sublattice was treated by means of the coherent potential approximation alloy theory (Stocks *et al.*, 1979 ▸).

The temperature-dependent magnetic properties have been studied using Monte Carlo (MC) simulations based on the classical Heisenberg model. As soon as the Te atoms are non-magnetic (only very small induced magnetic moments are obtained in the DFT calculations for Te atoms) the simulations are performed accounting for only the magnetic moments located on Cr sites. According to the experimental structure data shown in Table 2 ▸, there are two types of Cr atoms belonging to two different sublattices, Cr1 (fully occupied) and Cr2 (partially occupied). The system under consideration was analyzed with a Hamiltonian,

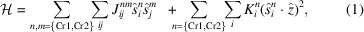

with unit vectors 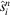 , 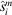 characterizing the direction of magnetic moments on sites *i*, sublattice *n* = {Cr1, Cr2} and sites *j*, sublattice *m* = {Cr1, Cr2}. The exchange-coupling 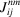 parameters were calculated using a scheme (Ebert & Mankovsky, 2009 ▸) which can be seen as a relativistic extension of the approach introduced by Liechtenstein *et al.* (1987 ▸). The constants of uniaxial magnetic anisotropy for Cr1 and Cr2 sublattices obtained within the magnetic torque calculations.

The MC simulations were performed for an infinite crystal, using the periodic boundary conditions applied to a supercell which consists of 9 × 9 × 9 crystallographic unit cells of the compound. The positions for the Cr atoms were chosen on the basis of our previous work (Wontcheu *et al.*, 2008 ▸; Huang *et al.*, 2006 ▸), fully occupied for the Cr1 sublattice and randomly occupied for the Cr2 sublattice.

## 3. Results and discussion

### 3.1. Synthesis of a novel CrTe_3_ phase at extreme conditions of high pressure and temperature

**Figure 1:**
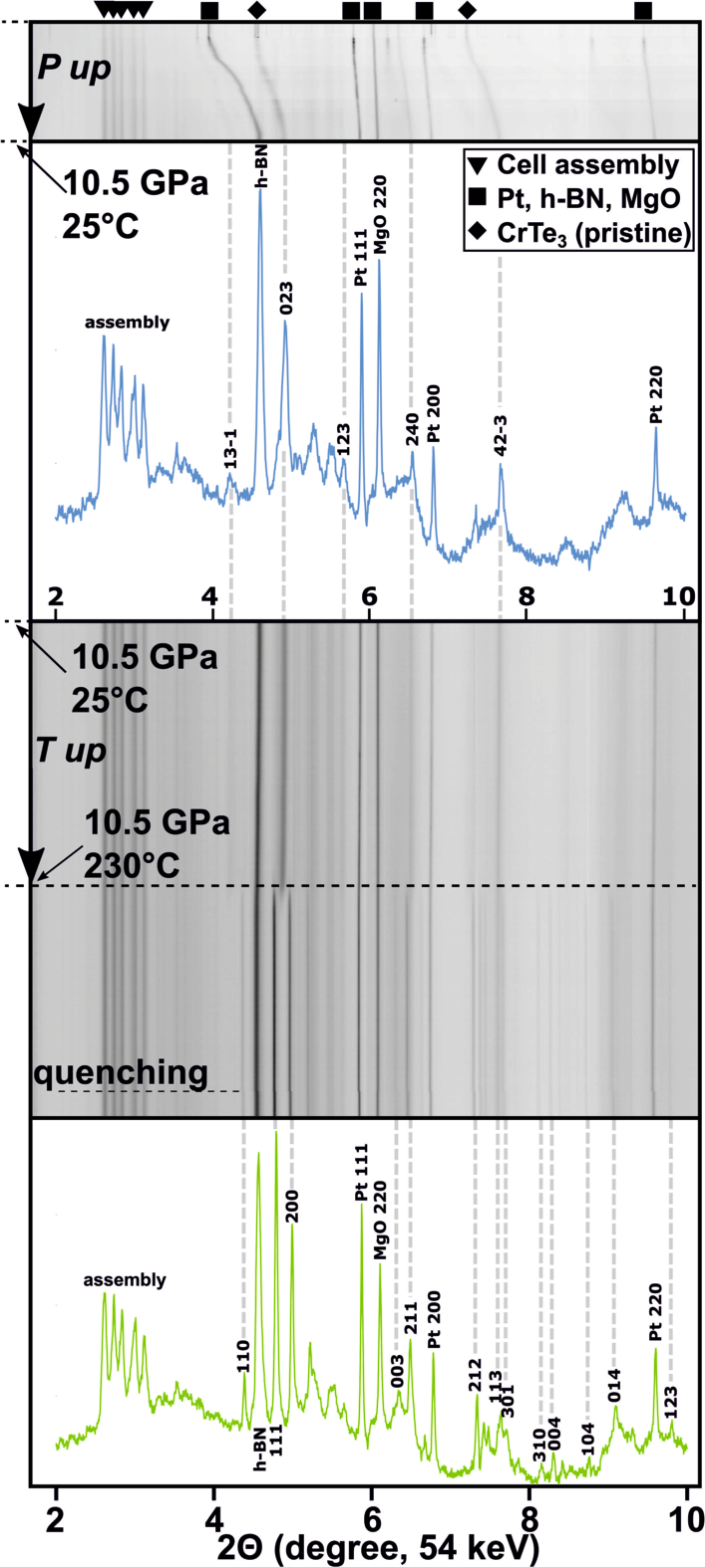
(*a*) Schematic compilation of diffractograms revealing the *in situ* phase transformation of the pristine CrTe_3_ phase to a novel high-pressure phase at 10.5 GPa and 230°C. Note that the shift of the reflection positions from the pristine CrTe_3_ and pressure markers to higher 2Θ values was caused by pressures exceeding several GPa.

High-*PT* *in situ* synchrotron X-ray diffraction experiments were performed to study the high-*PT* stability of CrTe_3_ at the ID06-LVP beamline at the ESRF. The crystal structure of the starting material was the monoclinic CrTe_3_ phase crystallizing in the space group *P*2_1_/*c*, which consists of edge-sharing CrTe_6_ octahedra along the *b*–*c* plane and van der Waals bonded layers perpendicular to the crystallographic *a* axis. Fig. 1 ▸ displays the high-*PT in situ* investigations that revealed a temperature-induced phase transformation of the pristine CrTe_3_ phase to a novel CrTe_3_ phase at a pressure of 10.5 GPa and a temperature of 230°C. The pristine CrTe_3_ phase remained stable during pressurization up to 10.5 GPa. Upon heating to 230°C, the strongest (023) reflection of the pristine CrTe_3_ in the 2D diffraction pattern around 4.9° (2Θ) clearly diminished, followed by the appearance of two new reflections at 4.8 and 5.0°. In addition, several further new reflections marked by the gray dashed lines were observed and assigned to the novel CrTe_3_ phase. Although the novel phase was stabilized under high pressures, the initial phase was recovered when slowly returning to ambient conditions. With all these observations in mind, the synthesis pressure was systematically changed to lower pressures to investigate pressure effects and fabricate larger samples for further characterization. By keeping the temperature at 250°C, the novel CrTe_3_ phase was also formed down to 6 GPa in additional *ex situ* syntheses.

### 3.2. Structure solution and refinement

First indexing attempts on *ex situ* synchrotron powder X-ray diffraction data resulted immediately in ortho­rhombic and monoclinic primitive unit-cell solutions, all exhibiting unit-cell volumes below 500 Å^3^. Most of these solutions gave acceptable whole powder pattern fits (Pawley, 1981 ▸), considering that some weak reflections might stem from impurity phases. One of the orthorhombic solutions had a rather high symmetry (space group *Pnn*2, volume ≃ 144 Å^3^); therefore, it was used for a first structural solution approach using the technique of simulated annealing. The simulated annealing converged quickly after a few dozen to a few hundred iterations into a Te-deficient crystal structure (chemical formula CrTe_2_), with the Cr atom located on a special position and the Te atom on a general position. The crystal structure motif consists of edge- and corner-sharing CrTe_6_ octahedra [see Fig. S6(*a*)], suggesting that the motif seems to be reasonable if a Te-deficient crystal structure is present. However, structure solution by a subsequent Rietveld refinement (Rietveld, 1969 ▸), using the fundamental parameter method (Rebuffi *et al.*, 2017 ▸) as implemented in *TOPAS*, failed. A detailed inspection of the fitted curve showed that the first two, rather weak, reflections must be considered as originating from an impurity phase, as the combination of space group and lattice parameters does not allow their modeling (see Fig. S2). However, it was not possible to match the additional peaks successfully to any phase. Moreover, other intensities and reflection profiles at higher diffraction angles were not satisfactorily modeled, although most of the resulting parameters were crystallographically in a reasonable range, except for a very high isotropic displacement parameter of the Cr atom. Since all observable reflections, except for one, can be explained by the same lattice parameters but reduced orthorhombic symmetry, other orthorhombic space groups and orthorhombic cells of larger size were considered for structural solution attempts. However, none of the approaches with orthorhombic models were successful. Supposing that the motif of the *Pnn*2 solution is somehow connected to the genuine solution, possible subgroups of the *Pnn*2 space group were explored using the *ISODISTORT* software (Campbell *et al.*, 2006 ▸). This approach revealed the monoclinic space group *P*2_1_ with a doubled unit-cell volume, which gave a quite good whole powder pattern fit. This agreement suggested that monoclinic space groups would be suitable candidates for the following structural solution attempts. Considering also higher symmetries of the found *P*2_1_ space group, a unit cell with space group *P*2_1_/*m* was used for further simulated annealing. Within a few thousand iterations a Te-rich crystal structure solution (chemical formula CrTe_4_) was found with a slightly different motif, as it consists now only of columnar ordered edge-sharing CrTe_6_ octahedra running along the crystallographic *b* axis [see Fig. S6(*c*)]. However, similar to the orthorhombic *Pnn*2 model, a subsequent Rietveld refinement revealed that this monoclinic solution cannot model accurately the observed diffraction pattern. The intensities of the weak reflections at low diffraction angles were clearly overestimated, while intensities and reflection profiles at higher diffraction angles were not satisfactorily modeled (see Fig. S3).

**Figure 2:**
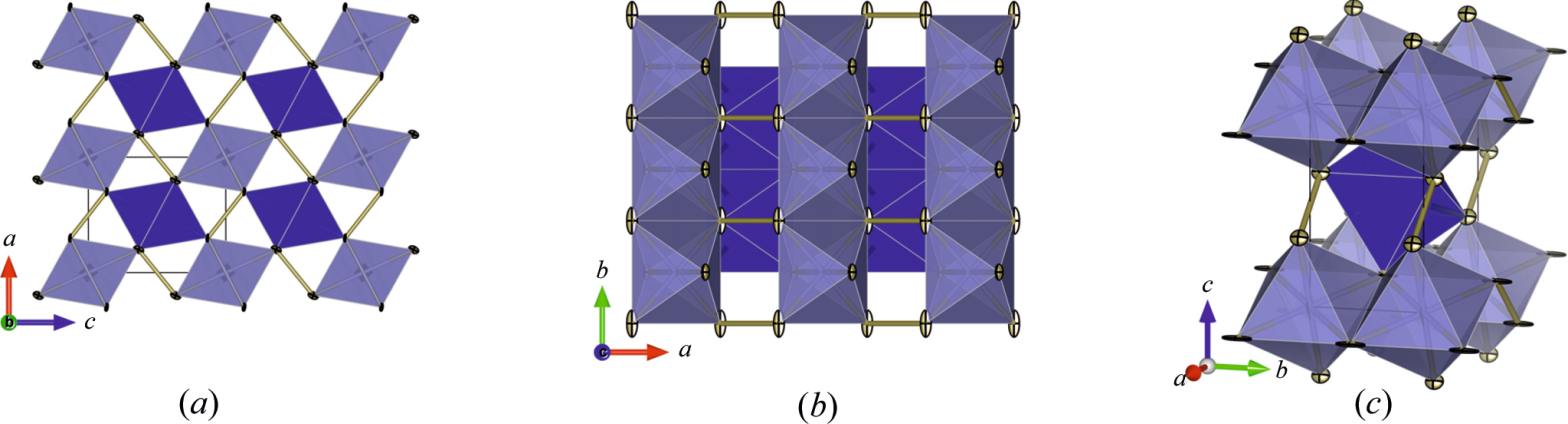
(*a*) Crystal structure of the quenched high-pressure phase of CrTe_3_ with space group *P*2/*m*. The fully occupied Cr-atom position is located in the center of the unit cell, while the Cr position with occupancy 1/3 is located at the corner position of the unit cell. Te–Te bonds are shown by the yellow lines. The corner-sharing motif can be best seen by a view along the crystallographic *b* axis (top). (*b*) The edge-sharing motif and columnar ordering of the CrTe_6_ octahedra can be best seen in projection along the crystallographic *c* axis. (*c*) Off-axis view close to the crystallographic *a* axis.

In addition, electron diffraction experiments indicated that the model in *P*2_1_/*m* of CrTe_3_ introduced systematic reflections in simulated PED patterns which were absent in the experimental diffraction patterns (not shown). Since the additional reflections in the simulated patterns may also be a hint that a higher-symmetry solution with fewer reflections was missed, a search for higher symmetry utilizing the *PLATON* software was undertaken (Spek, 2009 ▸). This search revealed that a monoclinic unit cell with half of the volume and space group *P*2/*m* could also be a possible solution (Fig. 2 ▸).

Although a subsequent Rietveld refinement of the X-ray data proved that most of the additional reflections compared with the *P*2_1_/*m* model (Fig. S3) were absent, the model still suffered from an overestimated intensity for the first weak reflections and poor intensity and peak profile modeling at higher diffraction angles (see Fig. S4).

Interestingly, all the above-described models include CrTe_6_ octahedra as building blocks, while they all suffer from an imperfect Rietveld refinement especially at higher diffraction angles. Consequently, another monoclinic model with the correct CrTe_3_ stoichiometry was proposed from electron diffraction data (see also Section 3.4). This crystal structure model shares the space group and lattice parameters of the *P*2*/m* model of CrTe_4_, except that an additional Cr position with an occupancy of 1/3 is added [see Fig. S6(*b*)]. Due to the additional partially occupied Cr position, a motif similar to that for the orthorhombic solution is obtained; more precisely, a corner-sharing network of the CrTe_6_ octahedra is established in the *a*–*c* plane, while a columnar ordering with edge-shared CrTe_6_ octahedra is established along the crystallographic *b* axis. The overall assembly of the CrTe_6_ octahedra resembles a deficient Marcasite-type structure with only every third cation position occupied within every second edge-sharing CrTe_6_ column.

**Figure 3:**
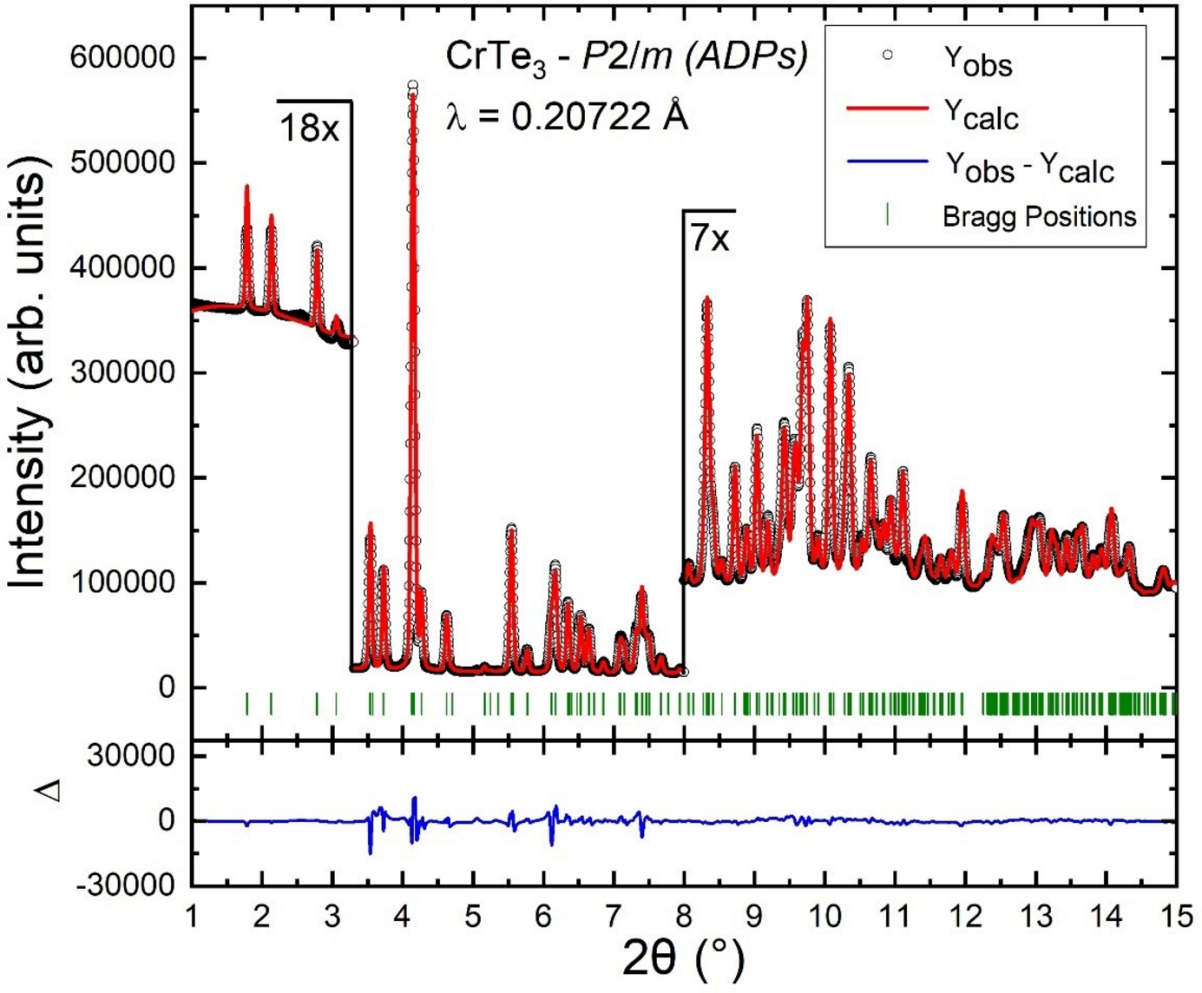
Powder X-ray diffraction data of a powder pellet of the novel CrTe_3_ phase measured at the Powder Diffraction and Total Scattering Beamline P02.1 (PETRA III/DESY). The shown fit of the Rietveld refinement is for the final crystal structure solution of the novel CrTe_3_ phase in space group *P*2/*m* with anisotropic displacement parameters for Cr and Te.

This stoichiometrically correct model was then refined with the Rietveld method (see Fig. S5). From this refinement, it became obvious that the weak reflections at low diffraction angle were now much better fitted, but that the reflections at higher diffraction angles still suffered from a poor fit. However, this could be overcome by changing isotropic to anisotropic displacement parameters for all atoms as depicted in Fig. 3 ▸. In this case, the Rietveld refinement improved drastically, which is seen not only in the difference curve but also by a drop of approximately 2.2% in the *R*_wp_ value (from 6.167 to 3.904%, *cf*. final Rietveld refinement shown in Fig. 3 ▸). We note that the requirement for anisotropic displacement parameters in this refinement may also reflect some kind of long-range disorder in the material.

### 3.3. Pair distribution function analysis

**Figure 4:**
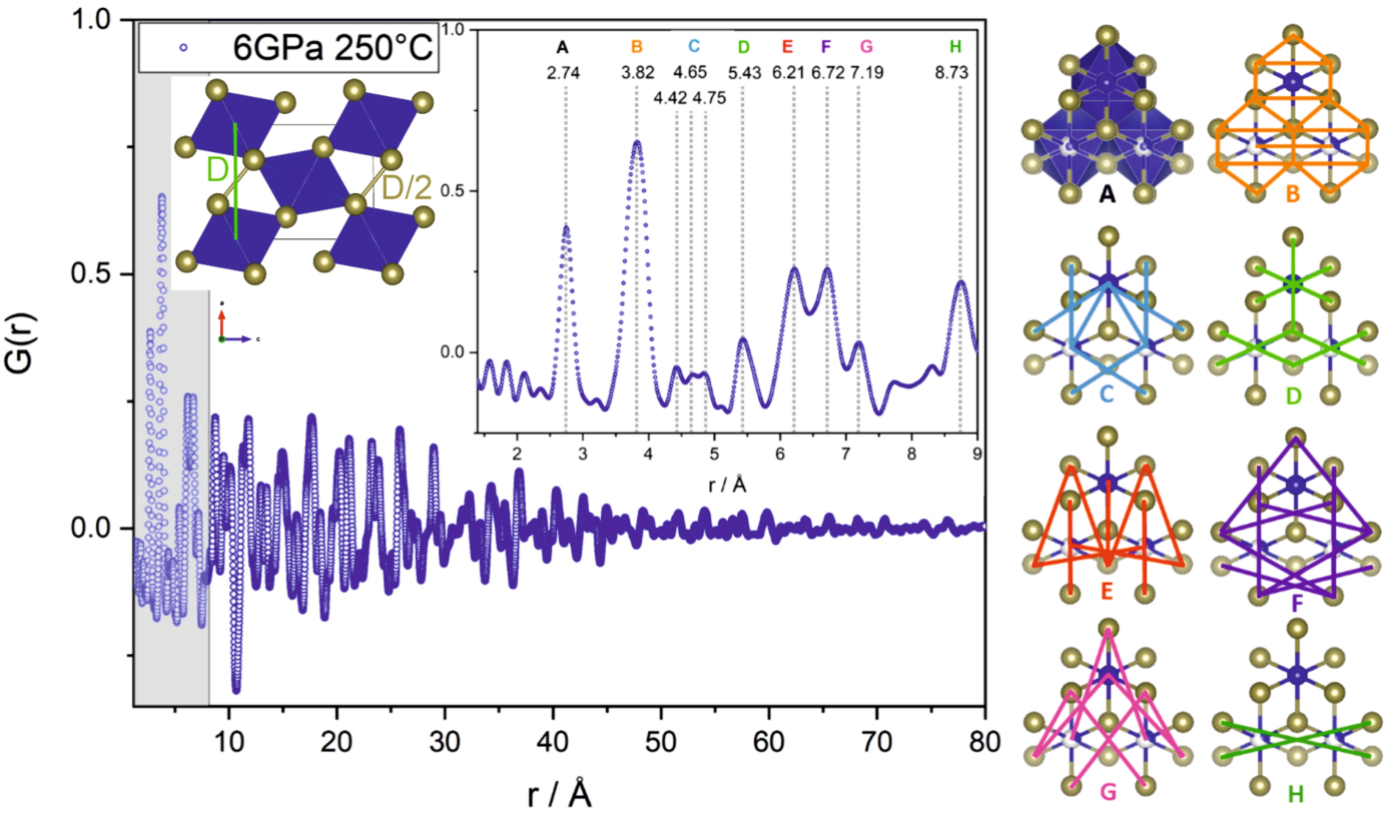
PDF with magnification of the local part *r* < 9 Å (gray area); all given distances are in Å. Corresponding distances of edge-sharing CrTe_6_ octahedra are depicted on the right (view onto the *b*–*c* plane). Peak A represents Cr–Te bonds of CrTe_6_ octahedra. Inset top left: schematic view onto the *a*–*c* plane. Included are Cr–Cr distances corresponding to peak D that could not be marked in the view on the right. Included in peak D are also short Te–Te distances interconnecting the chains of edge-sharing octahedra (yellow). They are short, only 2.78 Å (*r* ∼ D/2), indicating the presence of Te_2_^2−^ polyanions (Canadell *et al.*, 1992 ▸).

In Fig. 4 ▸, the PDF based on total scattering data of high-pressure CrTe_3_ is depicted. The PDF reveals a good crystallinity of the sample without any additional modulations or enhanced broadening of the peaks at high radius *r*. The strong damping of the function is caused by the small sample–detector distance and a consequent high *Q*_damp_ factor. The peaks at low *r* could all be explained by the local structural motifs of edge-sharing CrTe_6_ octahedra. An overview is given in Figs. 4 ▸ and 5 ▸.

**Figure 5:**
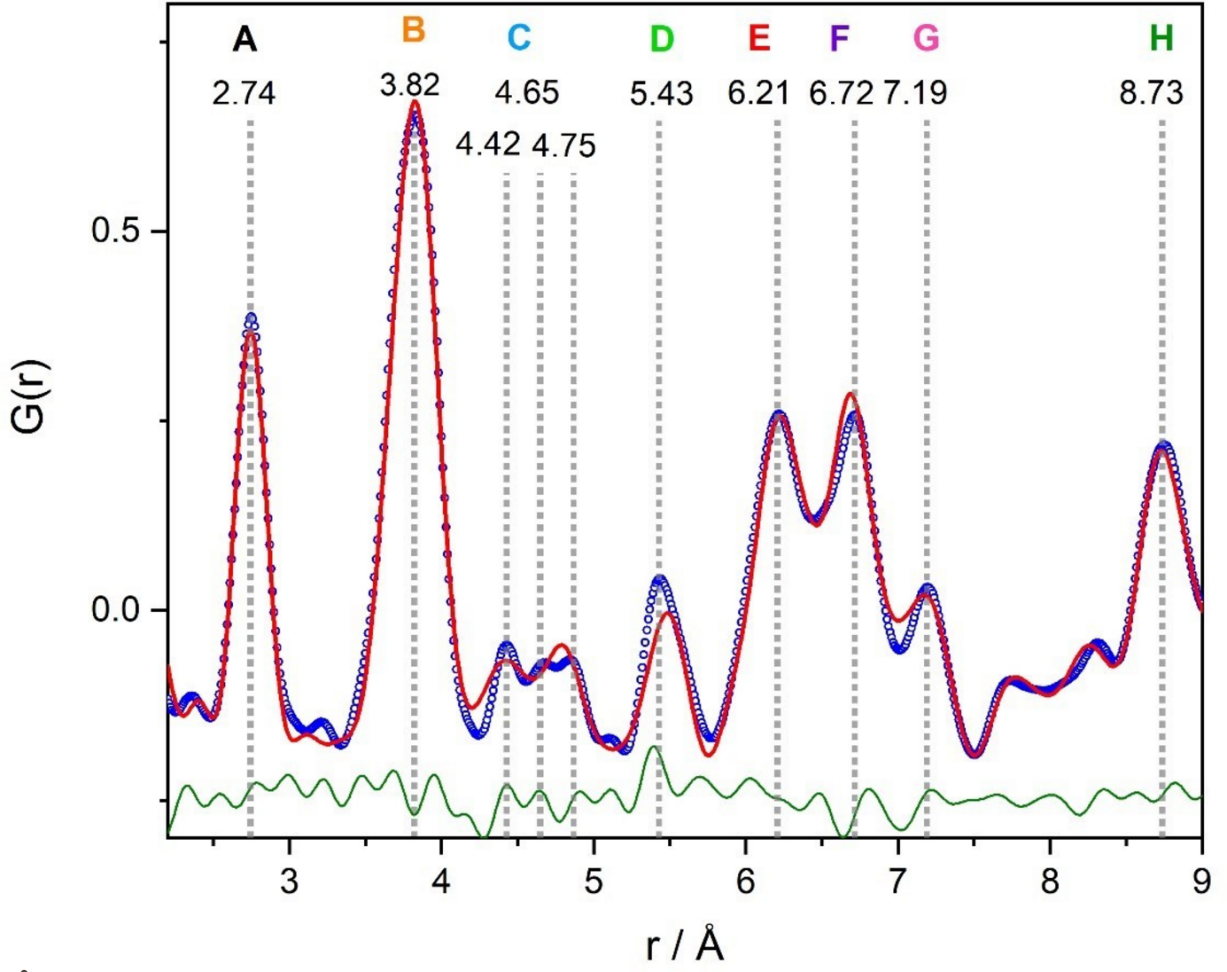
Real-space Rietveld refinement *G*(calc) of the *P*2/*m* structural model compared with the PDF data *G*(obs). The difference function *G*(diff) is displayed in green.

The real-space Rietveld fit (Fig. 5 ▸) agrees well with the observed PDF, indicating that the structural model derived by electron diffraction together with X-ray refinement as described in the previous section shares high coincidence with the observed real structure. The structural details of the refinement are summarized in Tables 3 ▸ and 4 ▸. The most obvious deviations of the modeled PDF are in the region of peaks C and D. Peak C corresponds to Cr–Te distances and peak D to doubled Te–Te distances of the Te_2_^2−^ polyanionic bond (2.78 Å, see Fig. 4 ▸) and Cr–Cr distances. Therefore, it is most likely that the deviation in the fit is due to two factors: the deviations could most likely indicate that chemical short-range ordering is present and the global statistical model does not fit perfectly, or they could be due to the unknown impurity phase, which was detected by XRD. To avoid overinter­pre­tation of the PDF data in the presence of an impurity, we did not investigate possible chemical short-range ordering. Never­theless, all the described deviations are small and the overall structural model fits very well. Keeping in mind that the material under investigation is an *ex situ* quenched high-pressure sample, which is prone to nanoscale inhomogeneities, TEM investigations are better suited to distinguish between local inhomogeneities and structural disordering.

### 3.4. Nanoscale analyses

#### 3.4.1. Chemical composition

The average stoichiometry of the as-synthesized crystalline powders of the pristine CrTe_3_ (*P*2_1_/*c*) and the quenched high-pressure phase of CrTe_3_ was examined by scanning electron microscopy (SEM)–EDX measurements. In this respect, the targeted 1:3 (Cr:Te) stoichiometry of CrTe_3_ is determined to be 1:2.95 on average for the starting compound and 1:2.92 for the quenched high-pressure phase (for individual measurements see Table S1 of the supporting information), which confirms an unchanged overall chemical composition. In order to examine possible local phase separation, *e.g.* into compounds with Cr:Te ratios of 1:2 and 1:4, which were initially supposed as possible solutions for the structure refinement, the chemical composition of individual microcrystals prepared as slices was examined by STEM–EDX. The distribution of Cr and Te across the microcrystals is presented in the elemental maps shown in Fig. S7. The evaluation of EDX spectra recorded from individual grains is summarized in Table S1 and shows local average stoichiometry ratios of 1:3.3 ± 0.3 which generally agrees with the nominal composition CrTe_3_, indicating just minute variations of the Cr-to-Te ratio. This finding excludes a segregation into CrTe_2_ and CrTe_4_ phases on the submicrometre scale.

At the grain boundaries we occasionally observed stronger segregations of Cr and Te restricted to the nanoscale, *cf*. EDX maps in Fig. S7, which do not affect the structure model of the novel and stoichiometric CrTe_3_ phase.

PED patterns were further compared with simulations of kinematic diffraction patterns using the refined structural models from synchrotron XRD experiments. Although local EDX measurements already excluded large-scale phase separation, such a comparison of experimental PED patterns with simulated patterns of the proposed models of CrTe_2_ and CrTe_4_ after first Rietveld refinements is presented in Fig. S8. There, the simulated patterns of CrTe_2_ show systematically absent reflections along the reciprocal directions [100]* for the examined [010] and [001] crystal orientations.

**Figure 6:**
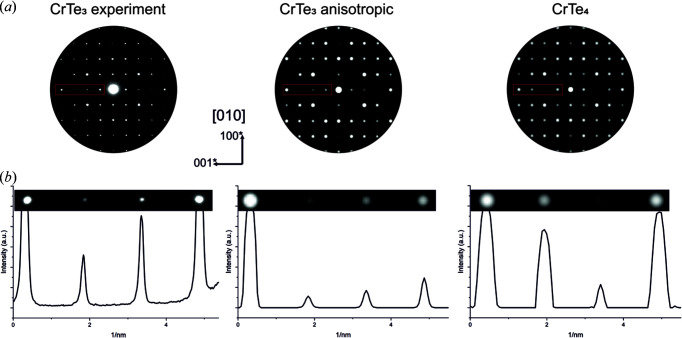
(*a*) Comparison of experimental and simulated [010] PED patterns using the proposed structure model for the high-pressure phase of CrTe_3_ (*P*2/*m*) with anisotropic displacement parameters and the CrTe_4_ (*P*2/*m*) phase. (*b*) Intensity profiles taken across the [001]* reflections marked in the red frame in part (*a*); left, experiment; middle, simulation; right, CrTe_4_.

The simulation of PED patterns for the CrTe_4_ (*P*2/*m*) model results in an acceptable agreement with the experimental PED patterns; however, subtle differences in the reflection intensity distribution, *e.g.* along the [001]* direction, are observed (compare Fig. 6 ▸). The lattice parameters and space group (*P*2*/m*) of the CrTe_4_ model provide a first approximation to the novel CrTe_3_ high-pressure phase, but further modification is required to adjust the Cr:Te stoichiometry and improve the fit of the simulation to the PED reflection intensity distribution. In our proposed structure model in *P*2/*m* a shift of all atom positions by (0, 0, *c*/2) is introduced, and one additional Cr position with an occupancy of 1/3 is added to the origin of the unit cell to achieve 1:3 stoichiometry [see Fig. S6(*b*)]. As discussed in Section 3.2, the proposed monoclinic model provided an improved fit to the X-ray diffractograms, especially after introducing anisotropic displacement parameters. This strong coincidence between the experiment and simulation using the proposed structure model is also observed when comparing the reflection intensity distribution of the simulated [010] PED pattern along the direction [001]* as shown in Fig. 6 ▸.

As a side note, traces of the initial monoclinic phase CrTe_3_ (*P*2_1_/*c*) were identified in electron diffraction measurements which could indicate a partially incomplete reaction or some transition via metastability during quenching from high pressures and high temperatures (see Fig. S9). These traces were only found in the TEM PED experiments. In the XRD and PDF no residuals of the initial CrTe_3_ were found and there was no match to the impurity peaks. For the XRD and PDF measurements the amount is too low, below 5%, to be detected. Even for the synchrotron, with a lower detection limit, no traces were found.

**Figure 7:**
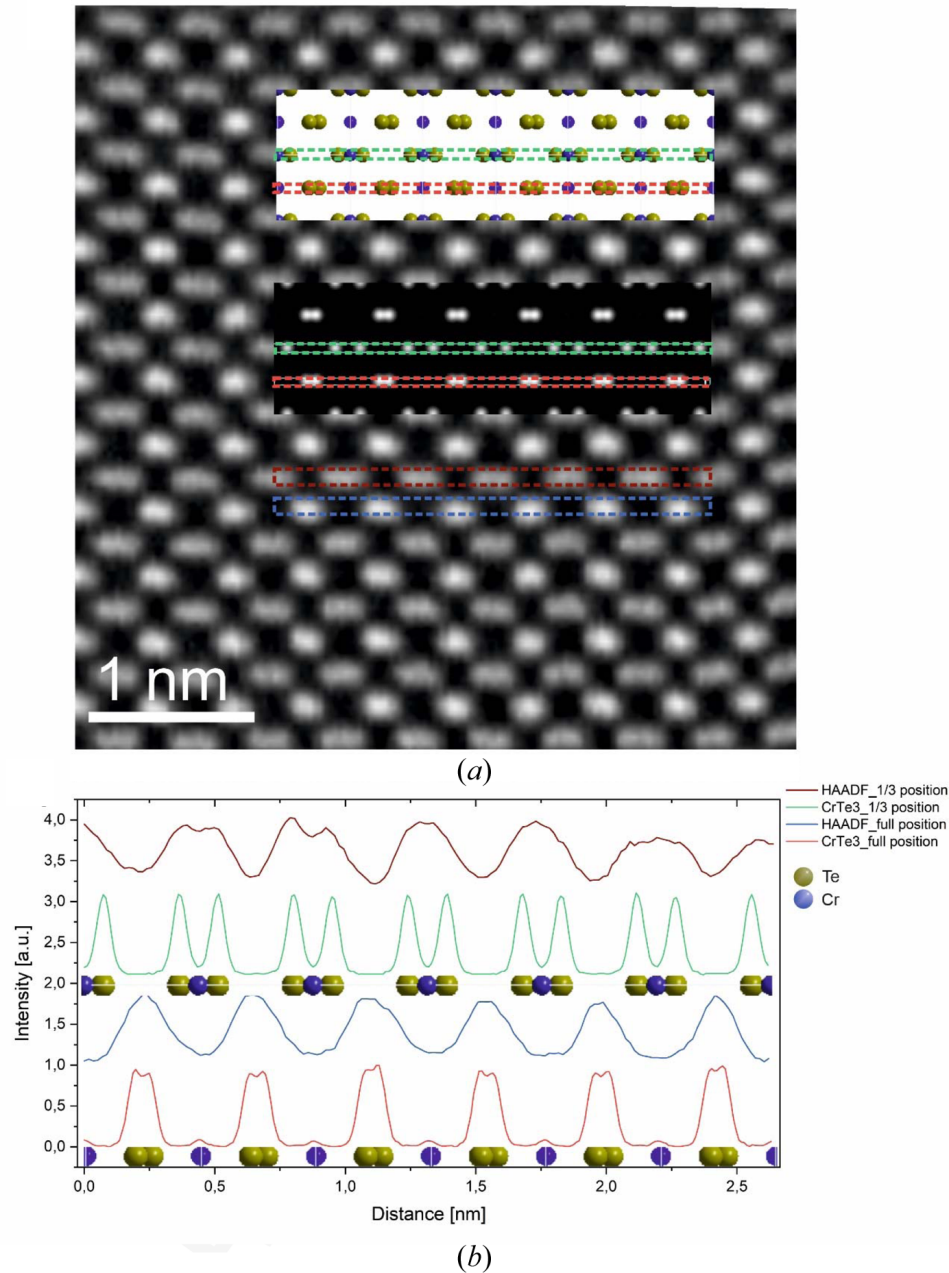
(*a*) Atomic resolution STEM image of CrTe_3_ (*P*2/*m*) in the [101] direction with an overlay of the corresponding atom positions and the simulated HAADF-STEM image. (*b*) Intensity profiles from the highlighted regions in the simulated and experimental images.

In order to collect additional evidence to verify the proposed structural model, atomic resolution STEM micrographs were recorded of grains depicted in Fig. S7. Fig. 7 ▸ shows a HAADF-STEM micrograph in which the atomic column positions of the Te sublattice are probed in the [101] orientation, in close agreement with the model structure [see top inset in Fig. 7 ▸(*a*)]. Model-based simulations of the atomic-number-dependent *Z*-contrast recorded using scattering collection angles of 80–200 mrad showed it was not possible to visualize the 1/3-occupied Cr positions (Cr2) along the [101] orientation. The intensity differences with respect to neighboring Te atoms [see bottom inset in Fig. 7 ▸(*a*)] displayed in the profiles across the STEM image and the simulated *Z*-contrast image in Fig. 7 ▸(*b*) are hardly significant. However, the expected double peaks [Fig. 7 ▸(*b*), green curve] are broadened experimentally (red curve), potentially due to positional dis­order of Te atoms interrelated with the occupational disorder of Cr2. Note that such disorder of Te is not included in the simulations.

Further, simulations for the [100] and [010] crystal orientations, which would allow a better view of the Cr atomic columns, demonstrated that the 1/3-occupied Cr columns cannot be differentiated in contrast to the strongly scattering Te atoms for both HAADF and annular bright-field modes (compare Fig. S10 in the supporting information). However, the presented STEM data provide additional nanoscale evidence for the proposed structure of the novel high-pressure CrTe_3_ phase with monoclinic space group *P*2/*m* and support PED data as well as the refined synchrotron diffraction data from macroscale powders.

### 3.5. Magnetic properties

**Figure 8:**
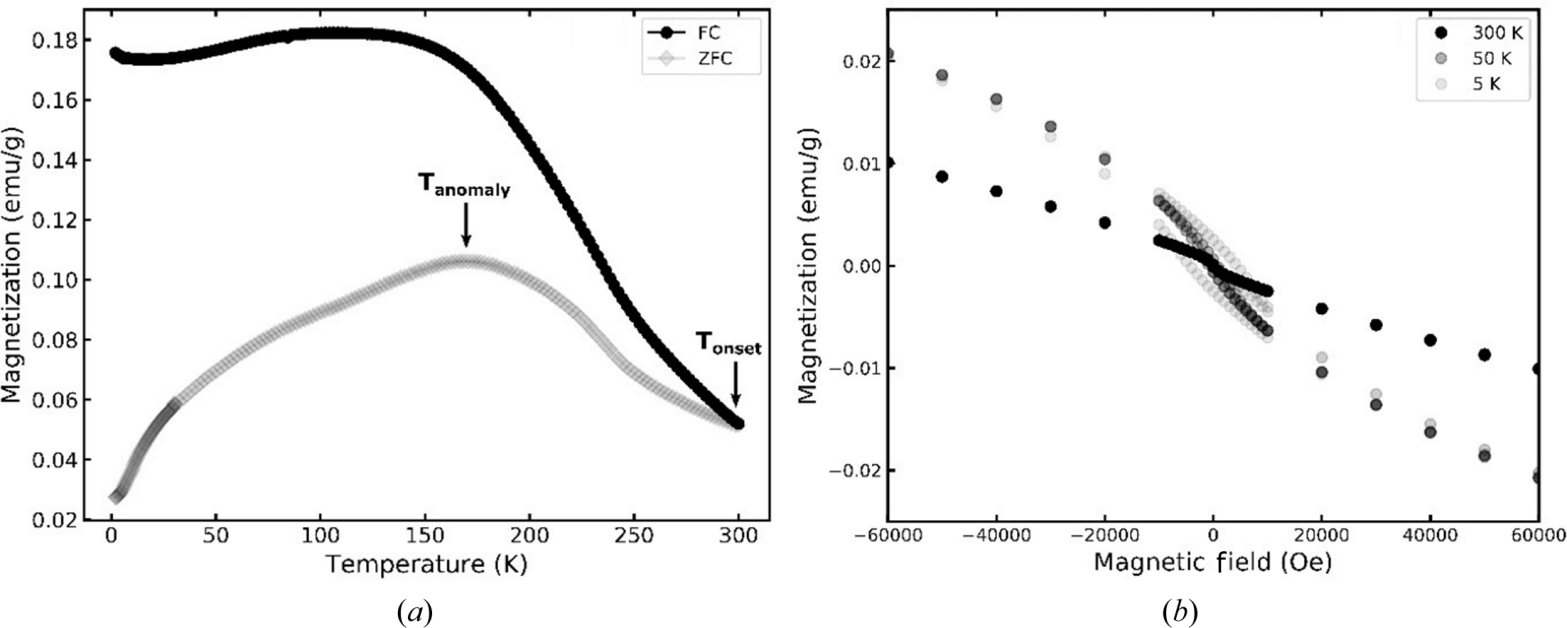
Magnetization versus temperature curves of a zero-field-cooled and field-cooled polycrystalline CrTe_3_ sample.

To probe the magnetic characteristics, temperature- and field-dependent magnetization (*M*) measurements were con­ducted. The temperature dependence of the magnetic moments was recorded in an applied field of 300 Oe under zero-field-cooled (ZFC) and field-cooled (FC) conditions. In Fig. 8 ▸, the ZFC and FC histories of the magnetic moments show a large variation between the *M*–*T* curves. The ZFC and FC curves start to separate from each other near room temperature because that is the temperature at which the warming was stopped and cooling started. The increase in magnetization as temperature decreases from room-temperature level aligns with the characteristics of local moment paramagnetism. The evolution of the ZFC and FC response typically resembles coexisting ferromagnetic (FM) and antiferromagnetic (AFM) clusters or classical spin glasses; details of the temperature dependence of the magnetic moments of the CrTe_3_ phase require further investigations. For other CrTe compounds both behaviors are observed as well. CrTe was calculated to transition from non-collinear to the ferromagnetic state at 30 K and from that to paramagnetic at 280 K (Polesya *et al.*, 2010 ▸). For the pristine CrTe_3_ phase AFM long-range ordering was observed below 55 K (McGuire *et al.*, 2017 ▸). The most plausible explanation for the magnetization curves is spin glass behavior, which corresponds to the tendency of Cr to form AFM interactions and the significant disorder present in the crystal structure. Further time-dependent DC magnetization measurements could offer more details. The variance in the curves, demonstrated by the more rapid increase of the FC data compared with the ZFC data, suggests different FM domains tend to neutralize each other (anti-align) after the zero-field cooling, whereas the domains align with field cooling. The field (*H*) dependence of the magnetic moments was investigated at three temperatures. The *M*–*H* data at the lowest temperature of 5 K feature a large hysteresis loop, indicating the presence of ferromagnetism in the CrTe_3_ compound. With increasing temperature, the magnitude of the hysteresis loop diminishes and it almost fully closes at room temperature. The latter finding is in accordance with the *M*–*T* data exhibiting no difference between ZFC and FC at room temperature.

### 3.6. Electronic structure calculations

**Figure 9:**
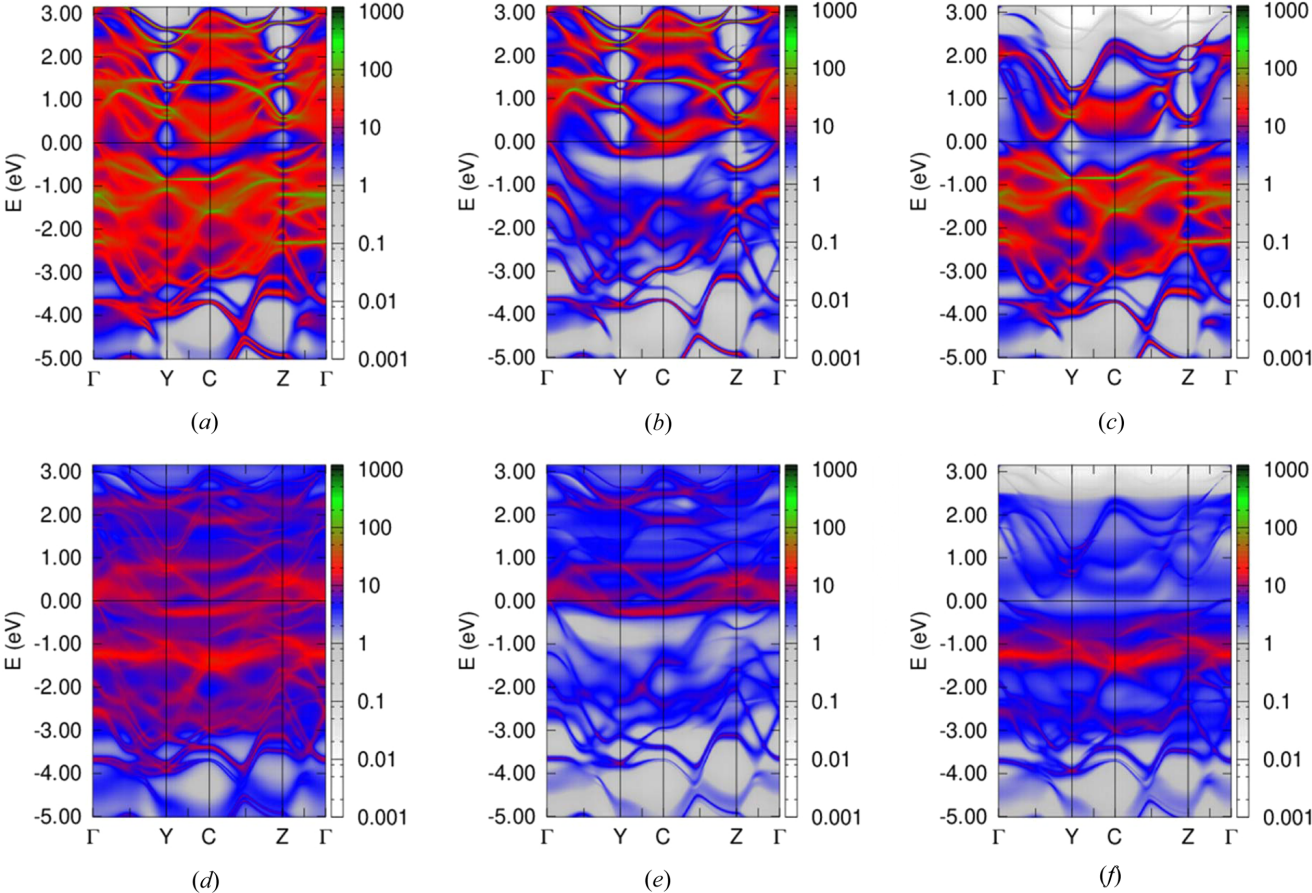
Element-projected spin-resolved BSF: total (*a*), (*d*), majority-spin (*b*), (*e*) and minority-spin (*c*), (*f*) states for Cr1 (top) and Cr2 (bottom) sublattices.

**Figure 10:**
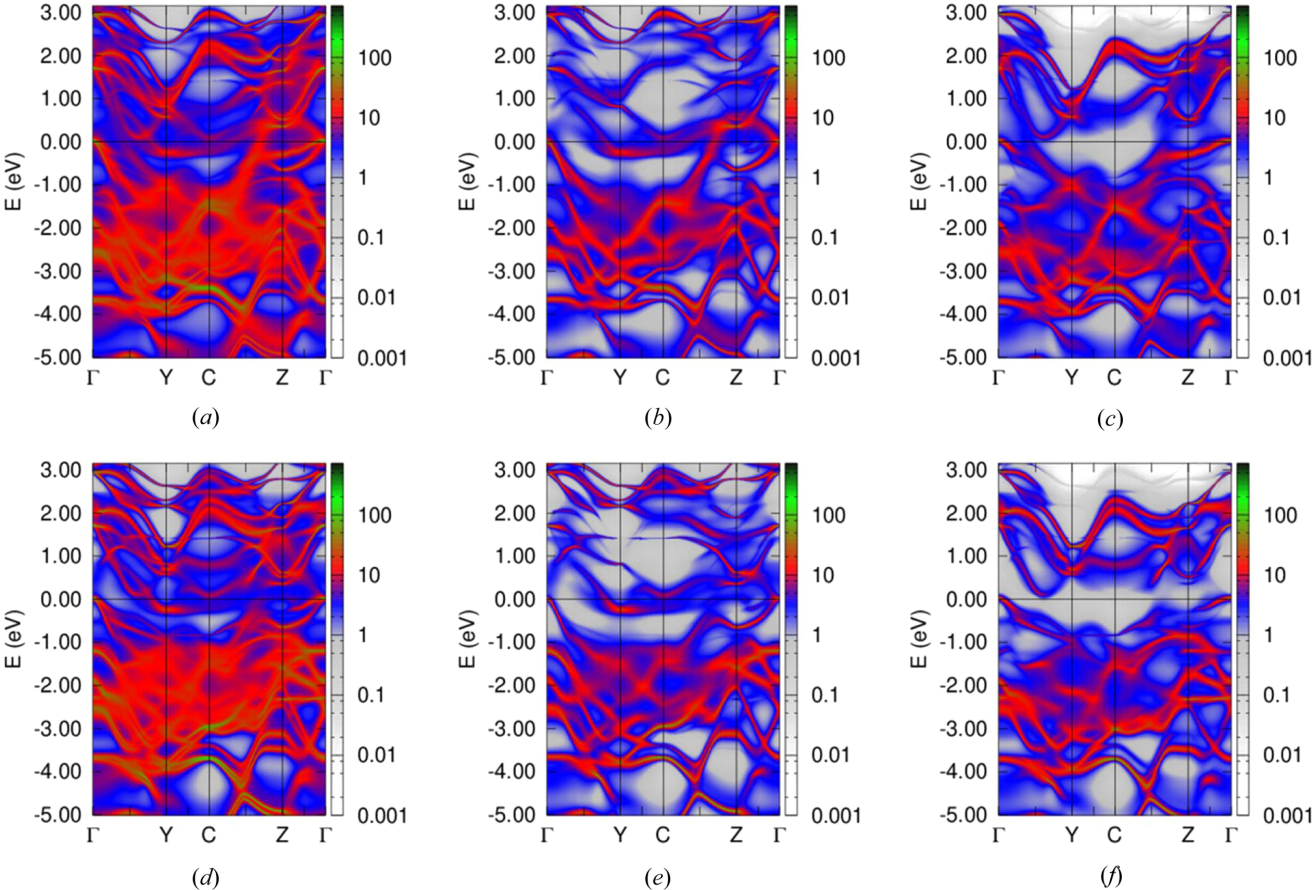
BSF for Te1 (top) and Te2 (bottom) atoms: total (*a*), (*d*), majority-spin (*b*), (*e*) and minority-spin states (*c*), (*f*).

Electronic structure calculations have been performed for the CrTe_3_ compound using the experimental structure data set. In this case, the Cr sublattice can be considered as a layered system composed of alternating fully and partially occupied Cr layers filled with Cr1 and Cr2 types, respectively. Also, two non-equivalent sites for the Te atoms, namely Te1 and Te2, have been distinguished. The element- and site-resolved electronic structures calculated for the FM state of the system are discussed below. Fig. 9 ▸ shows the Bloch spectral function (BSF) corresponding essentially to the con­ven­tional dis­persion relation *E*(*k*), but accounting for the sub-stoichiometry in the system, for Cr1 (Cr1 is fully occupied) (top) and Cr2 (bottom) sites: total (*a*) and (*d*), majority-spin (*b*) and (*e*), and minority-spin (*c*) and (*f*) states. Fig. 10 ▸ shows the BSF for Te1 and Te2.

**Figure 11:**
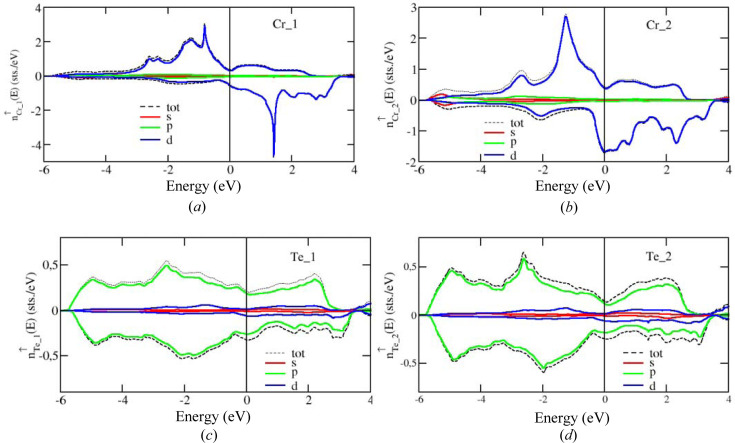
Spin- and orbital-resolved DOS on Cr1 (*a*), Cr2 (*b*), Te1 (*c*) and Te2 (*d*) sites.

**Figure 12:**
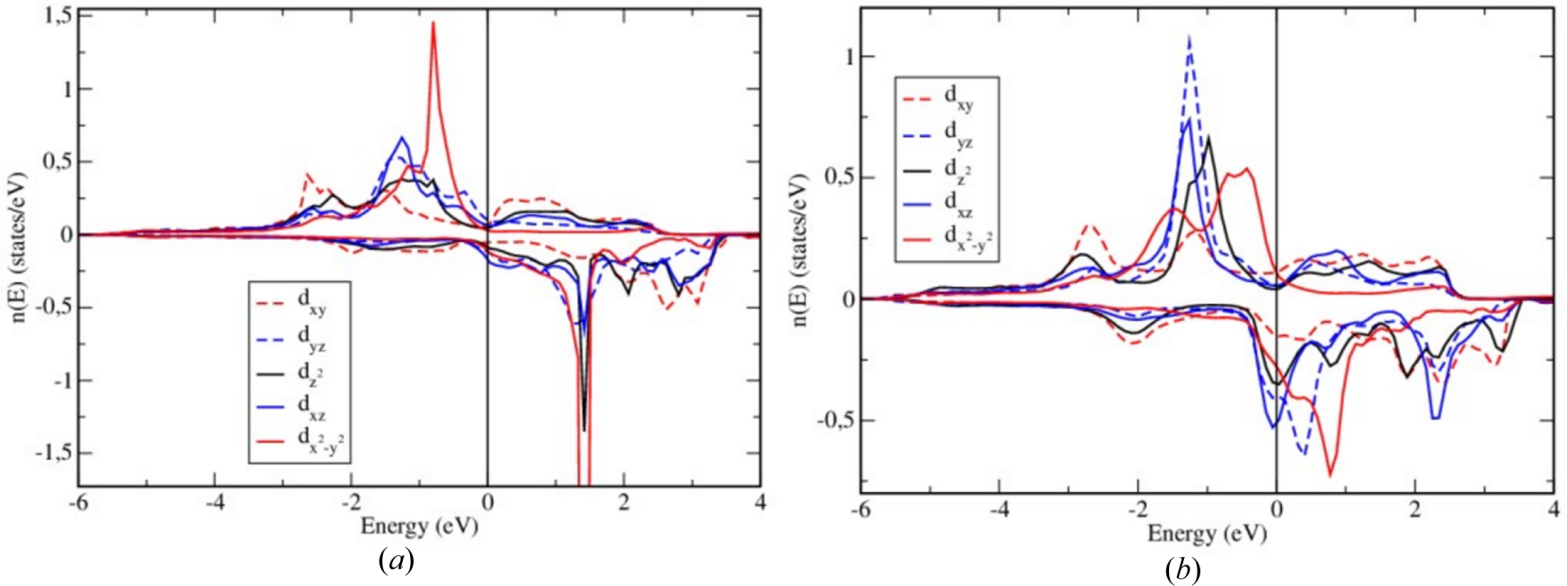
Spin- and (*l*, *m*)-resolved DOS on Cr1 (*a*) and Cr2 (*b*) sites.

The energy bands corresponding to the Cr2 sites are broadened in an appreciable way as a result of the chemical disorder within the partially occupied sublattice, while the bands corresponding to the fully occupied Cr1 sublattice are obviously less affected by the disorder in the Cr2 sublattice. This observation is also reflected by the Cr-projected density of state (DOS) shown in Figs. 11 ▸(*a*) and 11 ▸(*b*), with the Cr1 DOS having a more pronounced fine structure when compared with the Cr2 DOS. Although the Cr2 sublattice is incompletely occupied and, as a consequence, has a larger mean interatomic distance when compared with the fully occupied Cr1 sublattice, strong broadening of the Cr2 states due to disorder in the Cr2 sublattice leads to their band width being even larger than the band width of the Cr1 states. This effect is especially pronounced in the case of *d*_*x* ^2^__−*y* ^2^_ states which have a sharp DOS peak for the Cr1 sites, while this is essentially washed out for the Cr2 sites, as can be seen in the (*lm*)-resolved DOS shown in Fig. 12 ▸. Note, however, that the width of the *d_xz_*, *d_yz_* and *d*_*z* ^2^_ energy bands, which is essentially determined by the strong hybridization of these states with the states of the Te atoms, is comparable both for the Cr1 and Cr2 sublattices. Moreover, one can clearly see that the exchange splitting of the Cr1 majority- and minority-spin states is stronger than that of the Cr2 states. This results in the magnetic moment of the Cr1 atoms being equal to 2.81 µ_B_, which is much larger than that of the Cr2 atoms, 1.99 µ_B_. This effect can be partially attributed to the different charge transfer from the Te to the Cr1 and Cr2 atoms. The Cr2 atoms have about 0.6 electrons more than a neutral Cr atom, which is larger when compared with Cr1 atoms which have only about 0.15 extra electrons. While the number of majority-spin electrons for Cr1 and Cr2 is rather similar, different charge excess on Cr1 and Cr2 atoms results in a different occupation of minority-spin states for these atoms. As a consequence, the spin magnetic moment of Cr2 is smaller than that of the Cr1 atoms.

For the spin-resolved BSF corresponding to the Te1 and Te2 sublattices plotted in Fig. 10 ▸, one can also see a rather strong broadening of the energy bands. While there is no disorder present for the Te subsystem, both Te sublattices, Te1 and Te2, are adjacent to the Cr2 sublattice showing disorder. Thus, a broadening of the Te states is a consequence of the strong hybridization of the Te electronic states with the states of the Cr atoms randomly occupying the positions within the Cr2 sublattice.

The DOSs corresponding to the Te1 and Te2 sublattices are quite similar, with the difference ascribed to different coordination of the atoms Te1 and Te2: Te1 has one neighboring Cr1 atom and two Cr2 atoms, while Te2 is surrounded by one Cr2 atom and two Cr1 atoms. The induced spin moments on the Te atoms are −0.0356 and −0.0445 µ_B_ on Te1 and Te2, respectively.

**Figure 13:**
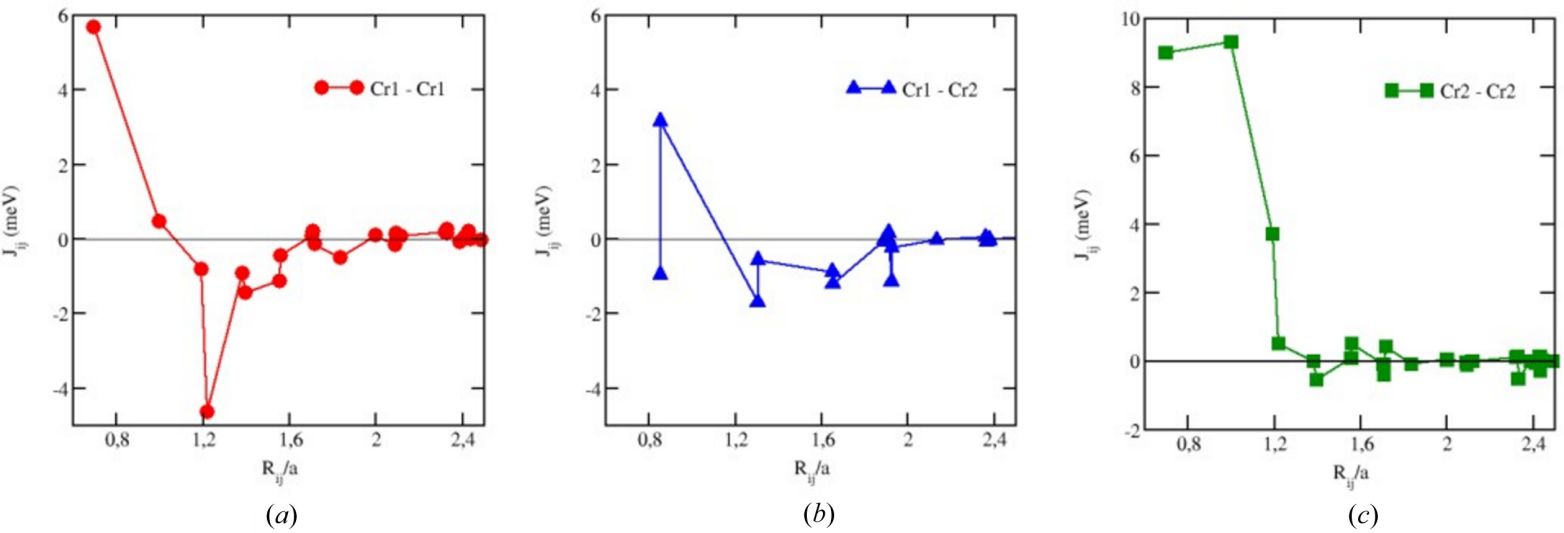
The Cr1–Cr1 (*a*), Cr1–Cr2 (*b*) and Cr2–Cr2 (*c*) exchange-coupling parameters.

To examine the ground-state magnetic structure and temperature-dependent magnetic properties, MC simulations were performed based on the Heisenberg Hamiltonian using the parameters for the exchange-coupling and the magneto-crystalline anisotropy (MCA) energy calculated from first-principles level. The MCA energy was calculated by making use of magnetic torque calculations (Staunton *et al.*, 2006 ▸), giving access to the difference in the total energy for states with two different directions of the magnetization: along the *x* (*E*_[100]_) and *z* (*E*_[001]_) axes, *i.e*. *E*_MCA_ = *E*_[100]_ − *E*_[001]_. These calculations led to a uniaxial MCA energy of 1.48 meV for Cr1 with the easy direction along the *z* axis, and 2.21 meV for Cr2 with the easy-plane direction, *i.e.* perpendicular to the *z* axis. The Cr–Cr exchange-coupling parameters are displayed in Fig. 13 ▸. As one can see, the nearest-neighbor Cr1–Cr1 exchange parameters *J_ij_* are positive, implying a favorable FM alignment of the spin magnetic moments of nearest-neighbor Cr atoms within the Cr1 sublattice. However, the interaction with the fourth-nearest neighbor is negative, *i.e.* it favors an AFM alignment of these spin moments. Moreover, these interactions are comparable in magnitude. As a consequence, competition of positive and negative exchange parameters together with the lattice structure can lead to a non-collinear magnetic structure within the Cr1–Cr1 sublattice. The Cr2–Cr2 interactions on the other hand are essentially positive, which should ensure an FM order within this sublattice. However, this partially occupied sublattice also couples with the Cr1 sublattice, adding another competing degree of freedom for the combined system. The resulting effect of all exchange interactions in the system is monitored by performing MC simulations, which are a well established tool to investigate both the ground state and temperature-dependent magnetic properties. Successful application of MC simulations was demonstrated previously in the particular case of Cr_*x*_(Te,Se)_*y*_ compounds, with both stoichiometric and non-stoichiometric Cr concentration (Wontcheu *et al.*, 2008 ▸; Polesya *et al.*, 2010 ▸, 2013 ▸).

**Figure 14:**
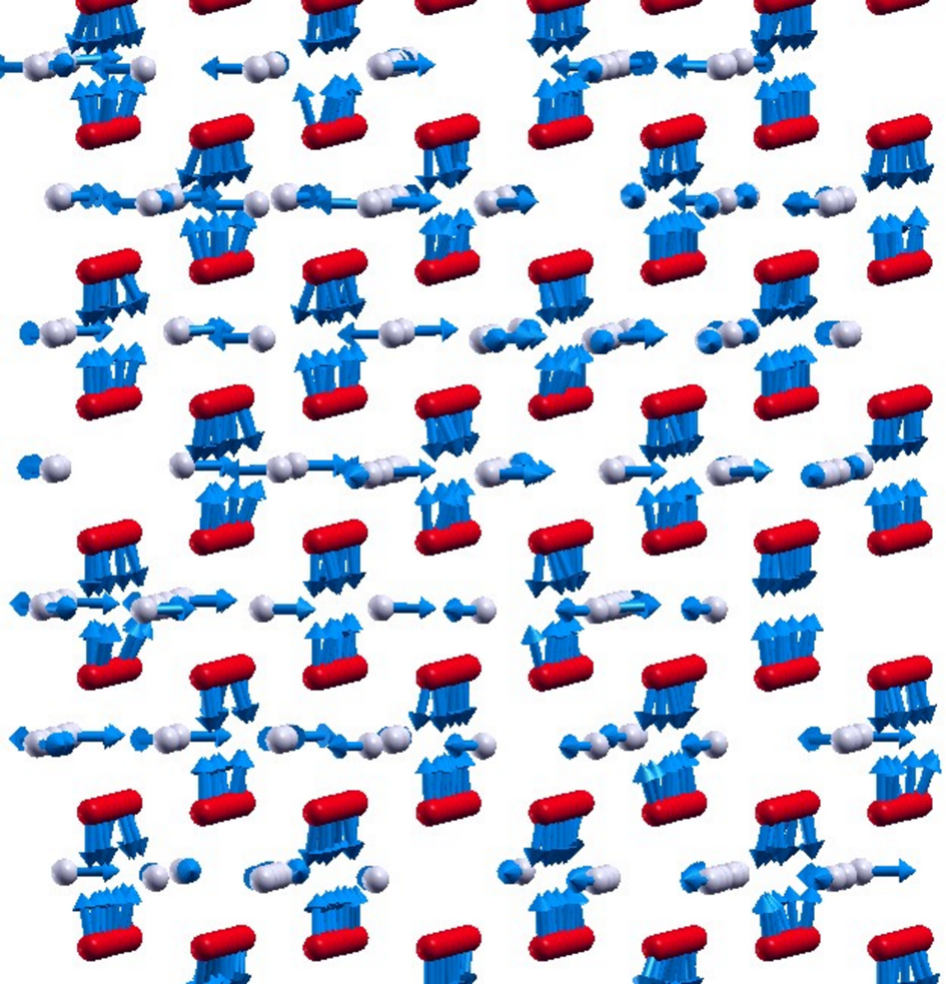
A snapshot of magnetic structure at *T* = 1 K, obtained within the MC simulations. Red: Cr1; gray: Cr2.

The system has a rather complicated magnetic structure at low temperatures (*T* = −272°C), which is displayed in Fig. 14 ▸. The magnetic moments of Cr1 are obviously ordered ferromagnetically within the atomic chains along the *y* direction, although their alignment is not perfect because of different atomic coordination at different Cr1 sites as a consequence of partial occupation of the Cr2 sublattice. On the other hand, one can see an AFM alignment of the Cr1 spin moments along the *x* and *z* directions, which leads to a zero total magnetic moment for this subsystem. The Cr2 atoms are arranged within the planes between the AFM-aligned planes of the Cr1 atoms, leading to a frustration concerning the orientation of the Cr2 magnetic moments. This is caused by competition between the interaction of similar strength with the neighboring Cr1 spin moments arranged within the layers above and below (along the *z* direction), which are aligned antiferromagnetically. At the same time, the Cr2 magnetic moments prefer to orient within the Cr2 layer due to the in-plane MCA obtained in DFT calculations.

The FM nearest-neighbor and next-nearest-neighbor interactions within the Cr2 sublattice do not play a significant role in the magnetic order in this sublattice because of incomplete occupation and the resulting increased mean interatomic distance. As a consequence, the Cr2 sublattice exhibits a random non-collinear magnetic structure. However, we stress the significant role played by MCA in the magnetic structure of the system, which results in a collinear alignment of the Cr1 spin magnetic and an in-plane orientation of all magnetic moments within the Cr2 sublattice. The critical temperature of the whole system is determined by the ordering transition in the Cr1 subsystem and was found to be ∼−138°C.

**Figure 15:**
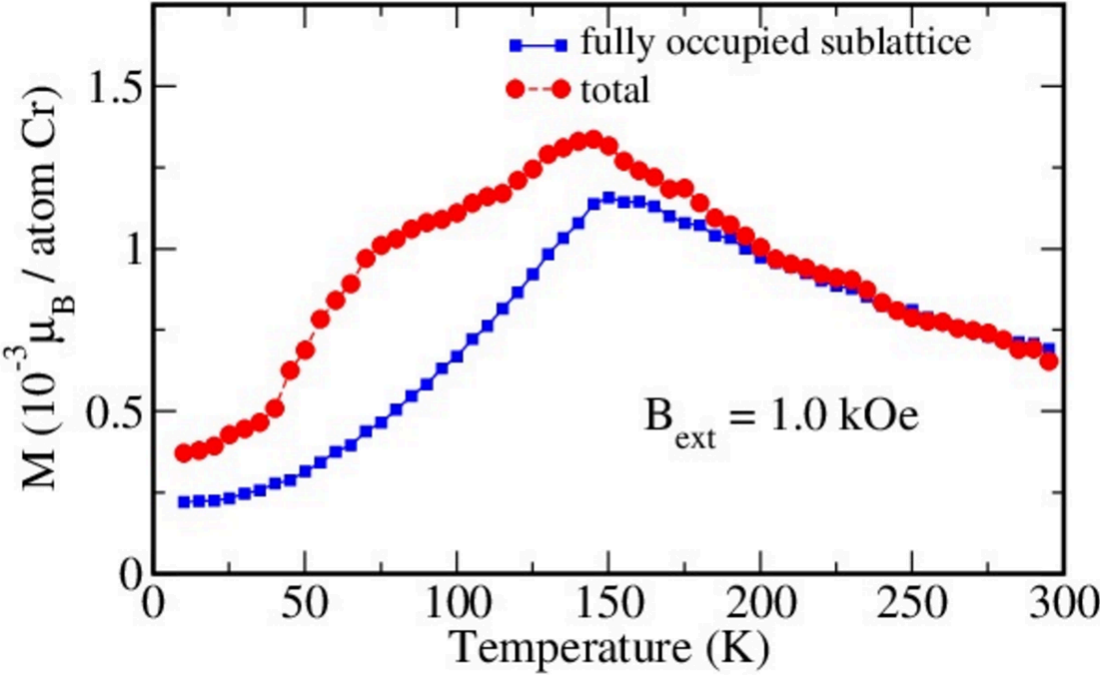
Results of Monte Carlo simulations: the magnetization (in 10^−3^ µ_B_ per Cr atom) as a function of the temperature in the presence of an external magnetic field oriented perpendicular to the Cr1 and Cr2 planes.

The temperature dependence of the magnetization obtained via MC simulations is shown in Fig. 15 ▸. The MC simulations have been performed for the system in the presence of an external magnetic field oriented perpendicular to the Cr1 and Cr2 planes. When the temperature decreases towards the critical one, *T*_N_ = 135 K (or −138°C), the magnetization increases, reaching a maximum value at the critical point. This behavior is a result of the increasing magnetic susceptibility of the magnetically disordered system in this temperature region (note that no magnetization increase occurs in the absence of an external magnetic field). However, below *T*_N_, the dominating AFM interactions within the Cr1 sublattice lead to a decrease of the magnetic moment in the Cr1 sublattice with decreasing temperature (shown by squares in Fig. 15 ▸). The net magnetic moment of the Cr2 sublattice also decreases with temperature, as a consequence of thermal disorder and the in-plane orientation of the Cr2 magnetic moments. These results are in good agreement with the experimental magnetization behavior observed for the ZFC regime, allowing the features of the experimental curve to be interpreted on the basis of our complementary theoretical calculations.

## 4. Conclusions

In conclusion, the structural solution of a novel CrTe_3_ phase using *ex situ* synchrotron powder X-ray diffraction data proved to be a challenging task. Although several structural models were obtained using simulated annealing, they all suffered from an imperfect Rietveld refinement, especially at higher diffraction angles. Finally, a stoichiometrically correct crystal structure model was proposed from electron diffraction data and refined using the Rietveld method. Additionally, the average stoichiometry of the synthesized monoclinic CrTe_3_ and the quenched high-pressure phase of CrTe_3_ was examined using SEM–EDX measurements. The targeted 1:3 (Cr:Te) stoichiometry of CrTe_3_ was determined to be 1:2.95 on average for the starting compound and 1:2.92 for the quenched high-pressure phase. Possible local phase separation into com­pounds with Cr:Te ratios of 1:2 and 1:4 was examined by STEM–EDX, which showed minute variations of the Cr-to-Te composition across multiple grains and the macroscopic average. Precession electron diffraction experiments were used for the nanoscale structure analysis of multiple grains from the quenched high-pressure phase. The proposed monoclinic model provided an improved fit to the X-ray diffractograms and the pair distribution function data. Atomic resolution STEM images were utilized to verify the proposed structural model. Traces of the initial monoclinic phase CrTe_3_ were also identified in electron diffraction measurements. With this information, the *P*2/*m* model was adjusted to the correct stoichiometry and occupancy of Cr atoms. The refinement was then drastically improved by changing the isotropic displacement parameters to anisotropic for all atoms, resulting in a good agreement between the calculated and experimental patterns. Additionally, the magnetic properties of the CrTe_3_ phase were investigated through temperature- and field-dependent magnetization measurements.

The results indicate the presence of AFM order in the system which is a result of the AFM interactions within the fully occupied Cr sublattice, as is shown with the DFT calculations. The magnetization of the partially occupied Cr sublattice with a random distribution of the Cr atoms is characterized by an in-plane orientation of the magnetic moments due to MCA and exhibits almost no magnetic order, as shown by the MC simulations. Nevertheless, the contribution of this sublattice to the finite temperature magnetization in the presence of an applied magnetic field is rather pronounced and magnetization is responsible for the low-temperature shoulder of the *M*(*T*) dependence. Finally, the maximum observed for the *M*(*T*) dependence can be attributed to the increasing magnetic susceptibility in the paramagnetic phase around the Neel temperature, leading to the increase of magnetization in the presence of a magnetic field. However, further investigations are needed to fully understand the magnetic properties of this material and find the reason for the anomaly.

## Supplementary Material

Crystal structure: contains datablock(s) I. DOI: 10.1107/S1600576724002711/te5131sup1.cif

Supporting information. DOI: 10.1107/S1600576724002711/te5131sup2.pdf

CCDC reference: 2343165

## Figures and Tables

**Table 1:** Crystal structure parameters for the final Rietveld refinement of the anisotropic displacement parameter model of the novel CrTe_3_ phase Residual values and goodness of fit (GoF) as defined in *TOPAS6.0* (Bruker, 2017 ▸).

Trivial name	Chromium telluride
Formula (nominal)	CrTe_3_
Crystal system	Monoclinic
Space group	*P*2/*m* (No. 10)
Lattice parameters (Å, °)	*a* = 5.5672 (2)
	*b* = 3.8834 (1)
	*c* = 6.6491 (2)
	ß = 90.074 (9)
Unit-cell volume (Å^3^)	143.75 (1)
Unit-cell mass (u)	579.73
Formula units, *Z*	1.333
*R*_exp_	0.61
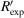	1.13
*R*_wp_	3.904
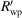	7.197
*R*_p_	2.803
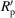	6.429
*R*_Bragg_	1.443
GoF	6.367

**Table 2:** Crystal structure parameters for the individual atomic positions for the final Rietveld refinement of the anisotropic displacement parameter model of CrTe_3_ Estimated standard deviations are given in parentheses.

Atom	*x*	*y*	*z*	Occupancy	Anisotropic displacement parameters (Å^2^)					
					*u*11	*u*22	*u*33	*u*12	*u*13	*u*23
Cr1	0.5	0.5	0.5	1	0.005 (5)	0.030 (8)	0.011 (5)	0	0.005 (5)	0
Cr2	0	0	0	1/3	0.000 (16)	0.000 (18)	0.006 (14)	0	0.024 (15)	0
Te1	0.3006 (3)	0.5	0.8688 (2)	1	0.008 (2)	0.055 (2)	0.001 (2)	0	0.001 (2)	0
Te2	0.1982(3)	0	0.3651 (3)	1	0.006 (2)	0.018 (2)	0.012 (2)	0	−0.003 (2)	0

**Table 3:** Crystal structure parameters for the final real-space refinement based on the model of CrTe_3_, derived from Rietveld refinement of PDF data Note that the monoclinic angle β was fixed during refinements due to the fact that PDF analysis is not the best method to determine cell angles.

Trivial name	Chromium telluride
Formula (nominal)	CrTe_3_
Crystal system	Monoclinic
Space group	*P*2/*m* (No. 10)
Lattice parameters (Å, °)	*a* = 5.542 (9)
	*b* = 3.87 (9)
	*c* = 6.6 (1)
	β = 90.074
Unit-cell volume (Å^3^)	143.4 (1)
*R*_wp_	10.94

**Table 4:** Crystal structure parameters for the individual atomic positions for the final real-space refinement based on the model of CrTe_3_, derived from Rietveld refinement of PDF data

Atom	*x*	*y*	*z*	Occupancy
Cr1	0.5	0.5	0.5	1
Cr2	0	0	0	1/3
Te1	0.30 (2)	0.5	0.87 (1)	1
Te2	0.19 (2)	0	0.36 (1)	1

## References

[bb1] Akram, M. & Nazar, F. M. (1983). *J. Mater. Sci.* **18**, 423–429.

[bb2] Barthel, J. (2018). *Ultramicroscopy*, **193**, 1–11. 10.1016/j.ultramic.2018.06.003 29906518

[bb3] Basham, M., Filik, J., Wharmby, M. T., Chang, P. C. Y., El Kassaby, B., Gerring, M., Aishima, J., Levik, K., Pulford, B. C. A., Sikharulidze, I., Sneddon, D., Webber, M., Dhesi, S. S., Maccherozzi, F., Svensson, O., Brockhauser, S., Náray, G. & Ashton, A. W. (2015). *J. Synchrotron Rad.* **22**, 853–858. 10.1107/S1600577515002283 PMC4416692 25931106

[bb4] Bensch, W., Helmer, O. & Näther, C. (1997). *Mater. Res. Bull.* **32**, 305–318.

[bb5] Billinge, S. J. L. & Farrow, C. L. (2013). *J. Phys. Condens. Matter*, **25**, 454202. 10.1088/0953-8984/25/45/454202 24140913

[bb48] Bruker (2017). *TOPAS6.0*. Bruker AXS, Madison, Wisconsin, USA.

[bb6] Buchner, M., Höfler, K., Henne, B., Ney, V. & Ney, A. (2018). *J. Appl. Phys.* **124**, 161101.

[bb7] Campbell, B. J., Stokes, H. T., Tanner, D. E. & Hatch, D. M. (2006). *J. Appl. Cryst.* **39**, 607–614.

[bb8] Canadell, E., Jobic, S., Brec, R. & Rouxel, J. (1992). *J. Solid State Chem.* **98**, 59–70.

[bb9] Chattopadhyay, G. (1994). *J. Phase Equilib.* **15**, 431–440.

[bb10] Chevreton, M., Bertaut, E. F. & Jellinek, F. (1963). *Acta Cryst.* **16**, 431.

[bb11] Chua, R., Zhou, J., Yu, X., Yu, W., Gou, J., Zhu, R., Zhang, L., Liu, M., Breese, M. B. H., Chen, W., Loh, K. P., Feng, Y. P., Yang, M., Huang, Y. L. & Wee, A. T. S. (2021). *Adv. Mater.* **33**, 2103360. 10.1002/adma.202103360 34477241

[bb71] Coelho, A. A. (2018). *J. Appl. Cryst.* **51**, 210–218.

[bb12] Coelho, A. A. (2000). *J. Appl. Cryst.* **33**, 899–908.

[bb13] Dijkstra, J., Weitering, H. H., Bruggen, C. F., Haas, C. & Groot, R. A. (1989). *J. Phys. Condens. Matter*, **1**, 9141–9161.

[bb14] Ebert, H., Ködderitzsch, D. & Minár, J. (2011). *Rep. Prog. Phys.* **74**, 096501.

[bb15] Ebert, H. & Mankovsky, S. (2009). *Phys. Rev. B*, **79**, 045209.

[bb73] Filik, J., Ashton, A. W., Chang, P. C. Y., Chater, P. A., Day, S. J., Drakopoulos, M., Gerring, M. W., Hart, M. L., Magdysyuk, O. V., Michalik, S., Smith, A., Tang, C. C., Terrill, N. J., Wharmby, M. T. & Wilhelm, H. (2017). *J. Appl. Cryst.* **50**, 959–966. 10.1107/S1600576717004708 PMC5458597 28656043

[bb16] Garcia, M. A., Fernandez Pinel, E., de la Venta, J., Quesada, A., Bouzas, V., Fernández, J. F., Romero, J. J., Martín González, M. S. & Costa-Krämer, J. L. (2009). *J. Appl. Phys.* **105**, 013925.

[bb70] Hammersley, A. P. (2016). *J. Appl. Cryst.* **49**, 646–652.

[bb17] Hansen, A.-L., Dietl, B., Etter, M., Kremer, R. K., Johnson, D. C. & Bensch, W. (2018). *Z. Kristallogr. Cryst. Mater.* **233**, 361–370.

[bb18] Jones, P. M., Rackham, G. M. & Steeds, J. W. (1977). *Proc. R. Soc. London A*, **354**, 197–222.

[bb44] Huang, M., Gao, L., Zhang, Y., Lei, X., Hu, G., Xiang, J., Zeng, H., Fu, X., Zhang, Z., Chai, G., Peng, Y., Lu, Y., Du, H., Chen, G., Zang, J. & Xiang, B. (2021). *Nano Lett.* **21**, 4280–4286. 10.1021/acs.nanolett.1c00493 33979154

[bb19] Huang, Z.-L., Bensch, W., Mankovsky, S., Polesya, S., Ebert, H. & Kremer, R. K. (2006). *J. Solid State Chem.* **179**, 2067–2078.

[bb20] Huang, Z.-L., Kockelmann, W., Telling, M. & Bensch, W. (2008). *Solid State Sci.* **10**, 1099–1105.

[bb21] Ipser, H., Komarek, K. L. & Klepp, K. O. (1983). *J. Less-Common Met.* **92**, 265–282.

[bb22] Ishizuka, M., Kato, H., Kunisue, T., Endo, S., Kanomata, T. & Nishihara, H. (2001). *J. Alloys Compd.* **320**, 24–28.

[bb23] Juhás, P., Davis, T., Farrow, C. L. & Billinge, S. J. L. (2013). *J. Appl. Cryst.* **46**, 560–566.

[bb24] Kanomata, T., Sugawara, Y., Kamishima, K., Mitamura, H., Goto, T., Ohta, S. & Kaneko, T. (1998). *J. Magn. Magn. Mater.* **177–181**, 589–590.

[bb25] Klepp, K. & Ipser, H. (1982). *Angew. Chem. Int. Ed. Engl.* **21**, 911.

[bb26] Klepp, K. O. & Ipser, H. (1979). *Monatsh. Chem.* **110**, 499–501.

[bb27] Kraschinski, S., Herzog, S. & Bensch, W. (2002). *Solid State Sci.* **4**, 1237–1243.

[bb28] Li, B., Deng, X., Shu, W., Cheng, X., Qian, Q., Wan, Z., Zhao, B., Shen, X., Wu, R., Shi, S., Zhang, H., Zhang, Z., Yang, X., Zhang, J., Zhong, M., Xia, Q., Li, J., Liu, Y., Liao, L., Ye, Y., Dai, L., Peng, Y., Li, B. & Duan, X. (2022). *Mater. Today*, **57**, 66–74.

[bb29] Li, C., Liu, K., Jiang, D., Jin, C., Pei, T., Wen, T., Yue, B. & Wang, Y. (2022). *Inorg. Chem.* **61**, 14641–14647. 10.1021/acs.inorgchem.2c01826 36067515

[bb30] Li, C., Liu, K., Jin, C., Jiang, D., Jiang, Z., Wen, T., Yue, B. & Wang, Y. (2022). *Inorg. Chem.* **61**, 11923–11931. 10.1021/acs.inorgchem.2c01659 35856941

[bb31] Li, H., Wang, L., Chen, J., Yu, T., Zhou, L., Qiu, Y., He, H., Ye, F., Sou, I. K. & Wang, G. (2019). *ACS Appl. Nano Mater.* **2**, 6809–6817.

[bb32] Li, R., Nie, J.-H., Xian, J.-J., Zhou, J.-W., Lu, Y., Miao, M.-P., Zhang, W.-H. & Fu, Y.-S. (2022). *ACS Nano*, **16**, 4348–4356. 10.1021/acsnano.1c10555 35191675

[bb33] Liechtenstein, A. I., Katsnelson, M. I., Antropov, V. P. & Gubanov, V. A. (1987). *J. Magn. Magn. Mater.* **67**, 65–74.

[bb34] Liu, Y., Abeykoon, M., Stavitski, E., Attenkofer, K. & Petrovic, C. (2019). *Phys. Rev. B*, **100**, 245114.

[bb35] Lukoschus, K., Kraschinski, S., Näther, C., Bensch, W. & Kremer, R. K. (2004). *J. Solid State Chem.* **177**, 951–959.

[bb36] McGuire, M. A., Garlea, V. O., Kc, S., Cooper, V. R., Yan, J., Cao, H. & Sales, B. C. (2017). *Phys. Rev. B*, **95**, 144421.

[bb37] Niu, K., Qiu, G., Wang, C., Li, D., Niu, Y., Li, S., Kang, L., Cai, Y., Han, M. & Lin, J. (2023). *Adv. Funct. Mater.* **33**, 2208528.

[bb38] Ohta, S., Kaneko, T. & Yoshida, H. (1996). *J. Magn. Magn. Mater.* **163**, 117–124.

[bb39] Oleynikov, P., Hovmöller, S. & Zou, X. D. (2007). *Ultramicroscopy*, **107**, 523–533. 10.1016/j.ultramic.2006.04.032 17291687

[bb40] Ozawa, K., Yoshimi, T., Irie, M. & Yanagisawa, S. (1972). *Phys. Status Solidi A*, **11**, 581–588.

[bb41] Pawley, G. S. (1981). *J. Appl. Cryst.* **14**, 357–361.

[bb42] Polesya, S., Kuhn, G., Benea, D., Mankovsky, S. & Ebert, H. (2013). *Z. Anorg. Allg. Chem.* **639**, 2826–2835.

[bb43] Polesya, S., Mankovsky, S., Benea, D., Ebert, H. & Bensch, W. (2010). *J. Phys. Condens. Matter*, **22**, 156002. 10.1088/0953-8984/22/15/156002 21389560

[bb45] Rebuffi, L., Sánchez del Río, M., Busetto, E. & Scardi, P. (2017). *J. Synchrotron Rad.* **24**, 622–635. 10.1107/S1600577517005434 28452754

[bb46] Rietveld, H. M. (1969). *J. Appl. Cryst.* **2**, 65–71.

[bb47] Spek, A. L. (2009). *Acta Cryst.* D**65**, 148–155. 10.1107/S090744490804362X PMC2631630 19171970

[bb49] Stadelmann, P. (2003). *Microsc. Microanal.* **9**, 60–61.

[bb50] Staunton, J. B., Szunyogh, L., Buruzs, A., Gyorffy, B. L., Ostanin, S. & Udvardi, L. (2006). *Phys. Rev. B*, **74**, 144411.

[bb51] Stocks, G. M., Temmerman, W. M. & Györffy, B. L. (1979). *Electrons in Disordered Metals and at Metallic Surfaces*, edited by P. Phariseau, B. L. Györffy & L. Scheire, pp. 193–221. Boston: Springer US.

[bb52] Tang, B., Wang, X., Han, M., Xu, X., Zhang, Z., Zhu, C., Cao, X., Yang, Y., Fu, Q., Yang, J., Li, X., Gao, W., Zhou, J., Lin, J. & Liu, Z. (2022). *Nat. Electron.* **5**, 224–232.

[bb53] Vainshtein, B. K. (2013). *Structure Analysis by Electron Diffraction*. Elsevier.

[bb54] Vosko, S. H., Wilk, L. & Nusair, M. (1980). *Can. J. Phys.* **58**, 1200–1211.

[bb55] Watanabe, N., Nagae, T., Yamada, Y., Tomita, A., Matsugaki, N. & Tabuchi, M. (2017). *J. Synchrotron Rad.* **24**, 338–343. 10.1107/S1600577516018579 PMC5182028 28009576

[bb56] Wen, Y., Liu, Z., Zhang, Y., Xia, C., Zhai, B., Zhang, X., Zhai, G., Shen, C., He, P., Cheng, R., Yin, L., Yao, Y., Getaye Sendeku, M., Wang, Z., Ye, X., Liu, C., Jiang, C., Shan, C., Long, Y. & He, J. (2020). *Nano Lett.* **20**, 3130–3139. 10.1021/acs.nanolett.9b05128 32338924

[bb57] Wontcheu, J., Bensch, W., Mankovsky, S., Polesya, S., Ebert, H., Kremer, R. K. & Brücher, E. (2008). *J. Solid State Chem.* **181**, 1492–1505.

[bb58] Yao, J., Wang, H., Yuan, B., Hu, Z., Wu, C. & Zhao, A. (2022). *Adv. Mater.* **34**, 2200236. 10.1002/adma.202200236 35419894

[bb59] Yuzuri, M., Kanomata, T. & Kaneko, T. (1987). *J. Magn. Magn. Mater.* **70**, 223–224.

[bb60] Zhang, J., Birdwhistell, T. & O’Connor, C. J. (1990). *Solid State Commun.* **74**, 443–446.

[bb61] Zhang, L.-Z., He, X.-D., Zhang, A.-L., Xiao, Q.-L., Lu, W.-L., Chen, F., Feng, Z., Cao, S., Zhang, J. & Ge, J.-Y. (2020). *APL Mater.* **8**, 031101.

[bb62] Zhang, L.-Z., Zhang, A.-L., He, X.-D., Ben, X.-W., Xiao, Q.-L., Lu, W.-L., Chen, F., Feng, Z., Cao, S., Zhang, J. & Ge, J.-Y. (2020). *Phys. Rev. B*, **101**, 214413.

[bb63] Zhang, X., Wang, B., Guo, Y., Zhang, Y., Chen, Y. & Wang, J. (2019). *Nanoscale Horiz.* **4**, 859–866.

[bb64] Zhao, D., Zhang, L., Malik, I. A., Liao, M., Cui, W., Cai, X., Zheng, C., Li, L., Hu, X., Zhang, D., Zhang, J., Chen, X., Jiang, W. & Xue, Q. (2018). *Nano Res.* **11**, 3116–3121.

