# Supplementary material for: Synthesis and in-depth structure determination of a novel metastable high-pressure CrTe3 phase
Source: J Appl Crystallogr. 2024 May 24;57(Pt 3):755–69. doi: 10.1107/S1600576724002711 (PMC11151671; doi:10.1107/S1600576724002711)
Supplement: Supplementary file 2 [file j-57-00755-sup2.pdf]

JOURNAL OF APPLIED CRYSTALLOGRAPHY

Volume 57 (2024)

Supporting information for article:

Synthesis and in-depth structure determination of a novel metastable high-pressure CrTe3 phase

Lennart Voss, Nico Alexander Gaida, Anna-Lena Hansen, Martin Etter, Niklas Wolff, Viola Duppel, Andriy Lotnyk, Wolfgang Bensch, Hubert Ebert, Sergey Mankovsky, Svitlana Polesya, Shrikant Bhat, Robert Farla, Masashi Hasegawa, Takuya Sasaki, Ken Niwa and Lorenz Kienle

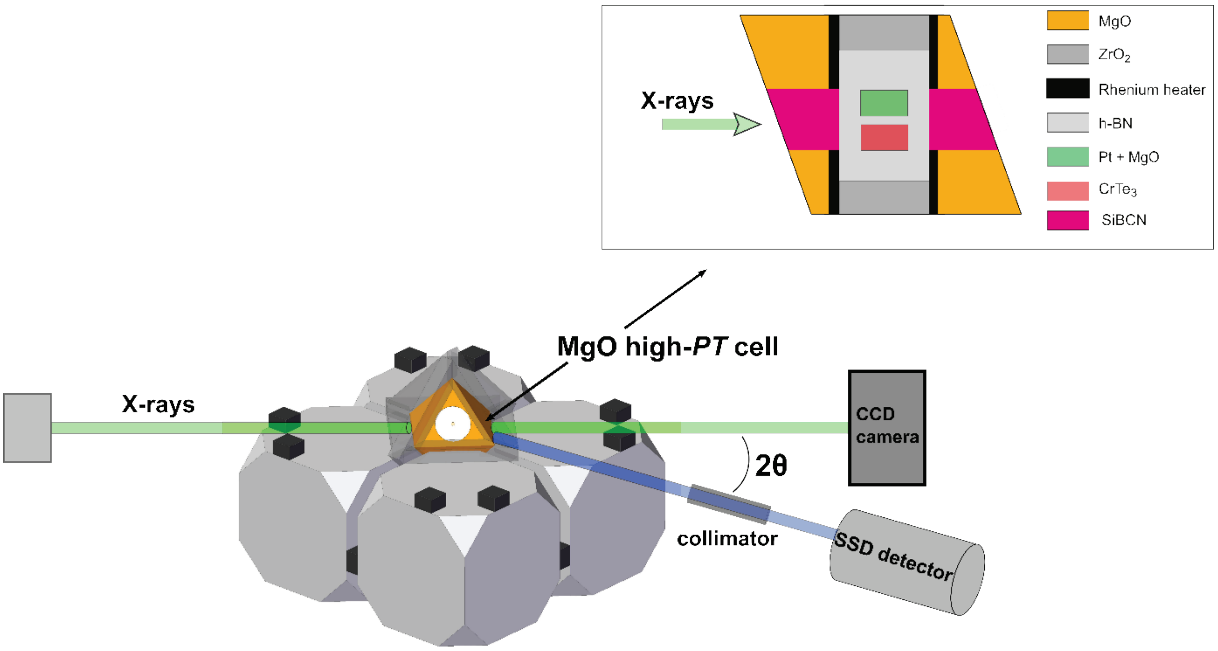

Figure S1: High-PT cell assembly applied for the in situ synchrotron diffraction experiments at the ESRF. (a) Cross section of the octahedral high-PT cell, in which Cr2O3-doped MgO was used as the pressure transmitting medium. (b) Configuration of the multi-anvil assembly consisting of WC anvils equipped with pyrophyllite gaskets with boron windows in beam direction. The WC anvils compress the octahedral pressure cell.

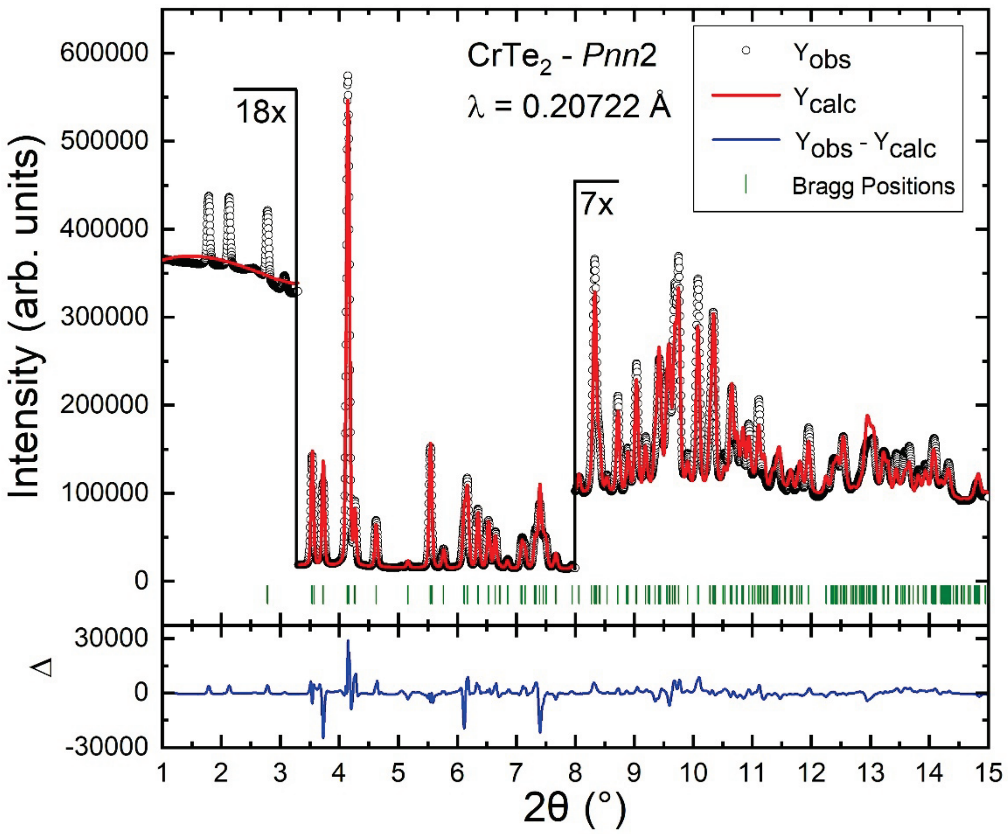

Figure S2: Powder X-ray diffraction data of a high pressure synthesized $CrTe_3$ powder pellet measured at the Powder Diffraction and Total Scattering Beamline P02.1 (PETRA III / DESY). The shown fit of the Rietveld refinement is a possible crystal structure solution in space group *Pnn*2 for a stoichiometry of $CrTe_2$. First reflections are not included for this crystal structure model and reflections at higher diffraction angles are not well fitted. R_{wp} for this model is 7.221 %.

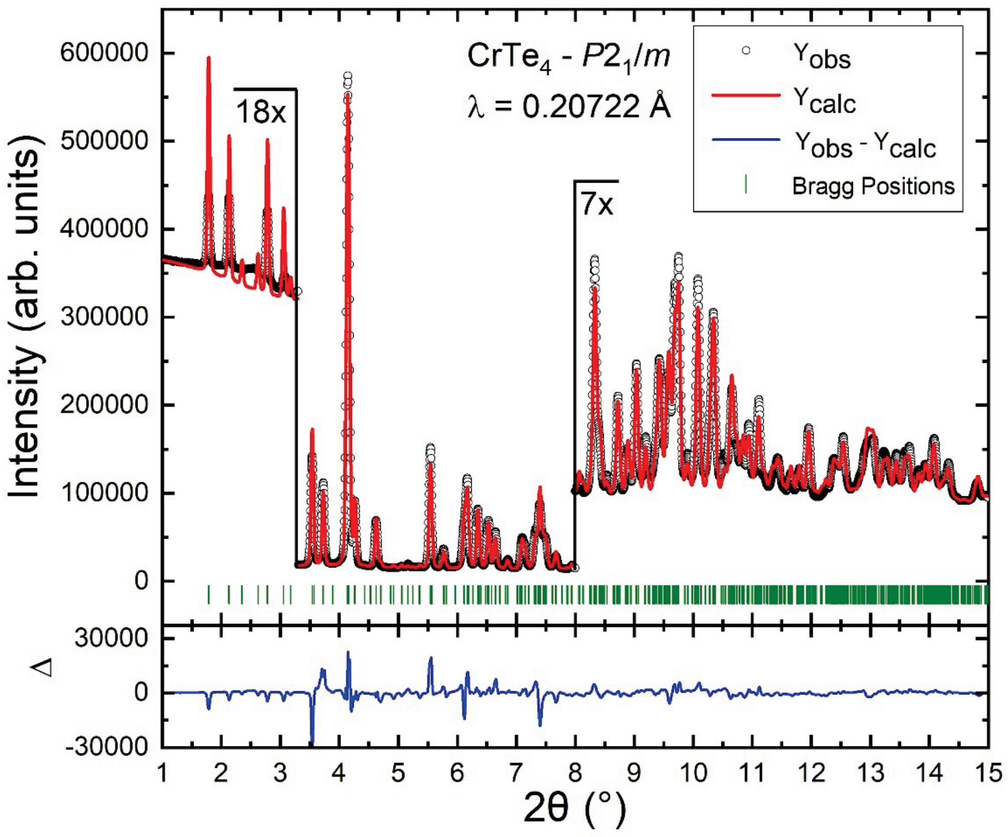

Figure S3: Powder X-ray diffraction data of a high pressure synthesized $CrTe_3$ powder pellet measured at the Powder Diffraction and Total Scattering Beamline P02.1 (PETRA III / DESY). The shown fit of the Rietveld refinement is a possible crystal structure solution in space group $P2_1/m$ for a stoichiometry of $CrTe_4$. The intensity of the first small reflections is clearly overestimated in addition to the reflections at higher diffraction angles which are not well fitted. Furthermore, the model creates intensity for reflections which are not observable in the experimental data. R_{wp} for this model is 7.300 %.

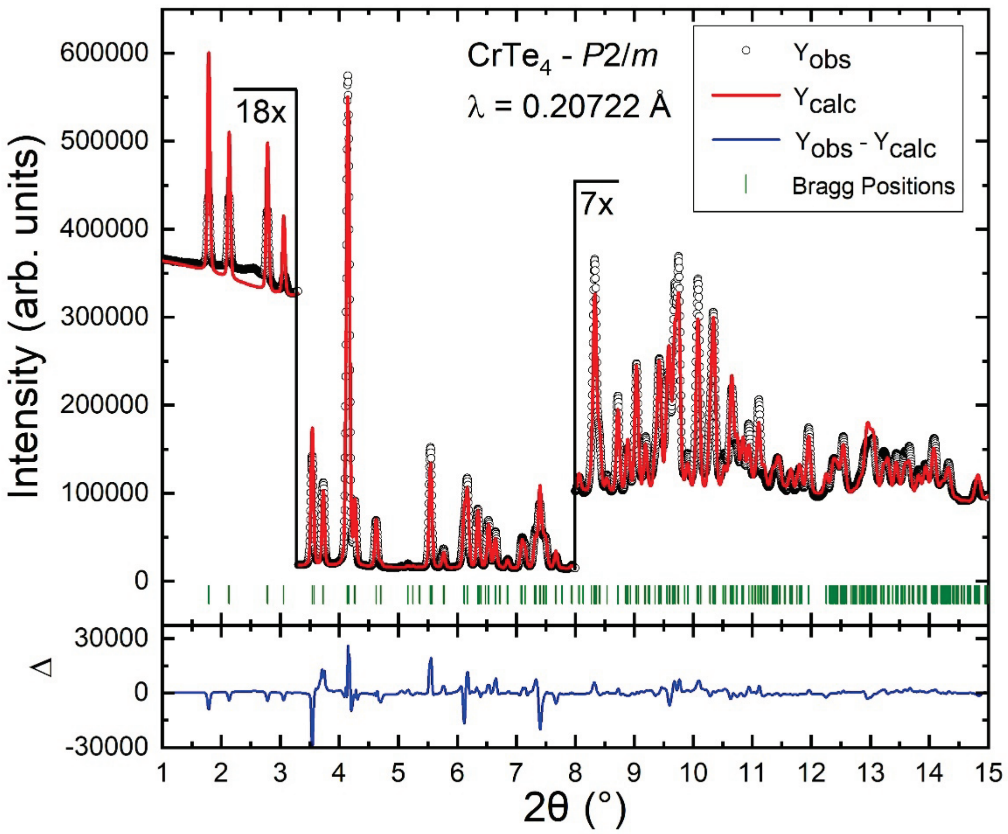

Figure S4: Powder X-ray diffraction data of a high pressure synthesized $CrTe_3$ powder pellet measured at the Powder Diffraction and Total Scattering Beamline P02.1 (PETRA III / DESY). The shown fit of the Rietveld refinement is a possible crystal structure solution in space group *P*2/*m* for a stoichiometry of $CrTe_4$. The intensity of the first small reflections is clearly overestimated in addition to the reflections at higher diffraction angles which are not well fitted. R_{wp} for this model is 7.651 %.

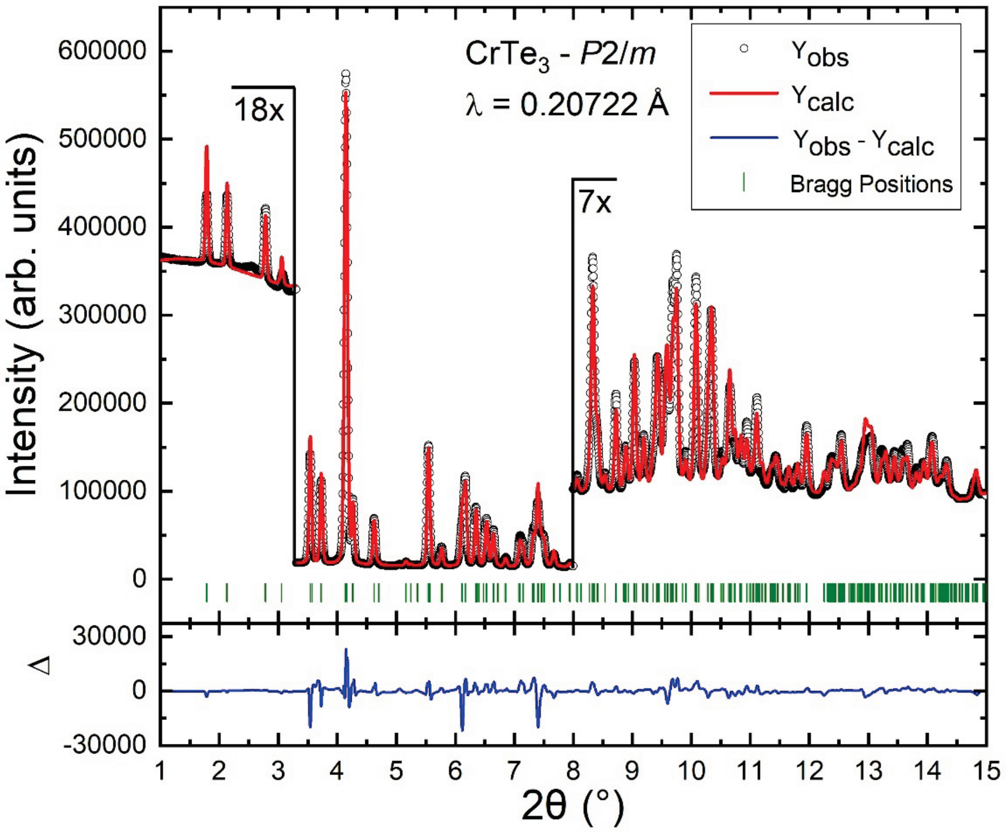

Figure S5: Powder X-ray diffraction data of a high pressure synthesized $CrTe_3$ powder pellet measured at the Powder Diffraction and Total Scattering Beamline P02.1 (PETRA III / DESY). The shown fit of the Rietveld refinement is the final crystal structure solution in space group *P*2/*m* with isotropic displacement parameters for the atomic positions for a stoichiometry of $CrTe_3$. The intensity of the first small reflections is slightly overestimated in addition to the reflections at higher diffraction angles which are not well fitted. R_{wp} for this model is 6.167 %.

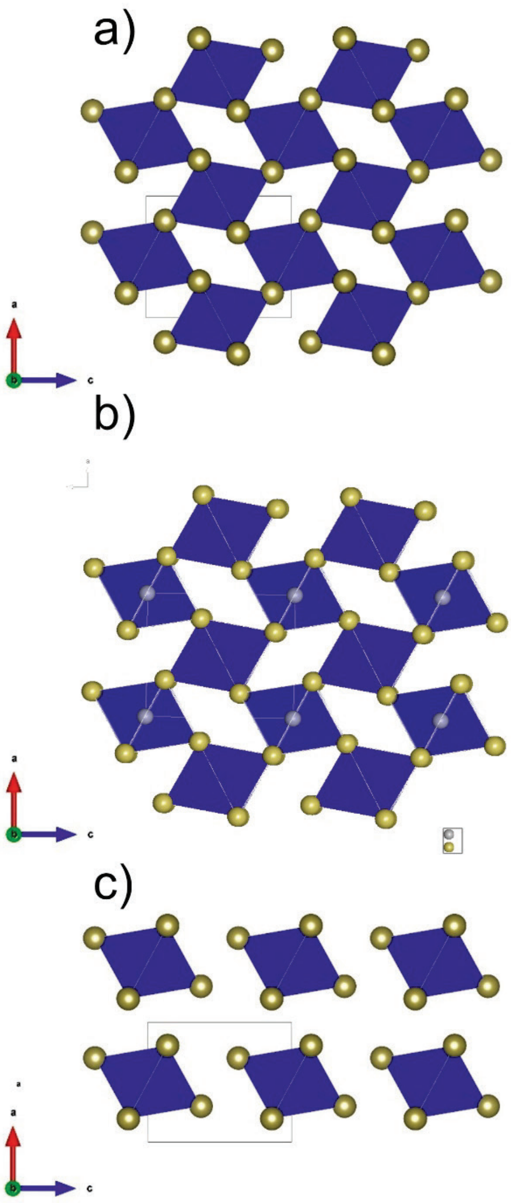

Figure S6: a) Possible crystal structure solution in space group *Pnn*2 for a stoichiometry of. Columns of edge-sharing $CrTe_6$ octahedra are interconnected by common corners. Stoichiometry of $CrTe_2$. b) Structure solution of $CrTe_3$ (*P*2/*m*). This structure represents the anisotropically refined solution of the partially filled $CrTe_4$ to fit the stoichiometry. The partially occupied Cr position is shown within semitransparent octahedra. The projection along the crystallographic *b*-axis shows the similarities to the $CrTe_2$ *Pnn*2. c) Possible crystal structure solution in space group *P*2/*m* for a stoichiometry of $CrTe_4$. This solution was derived from the $P2_1/m$ model, see text. A view along the crystallographic *b*-axis shows that there is no connection between the columns of $CrTe_6$ octahedra.

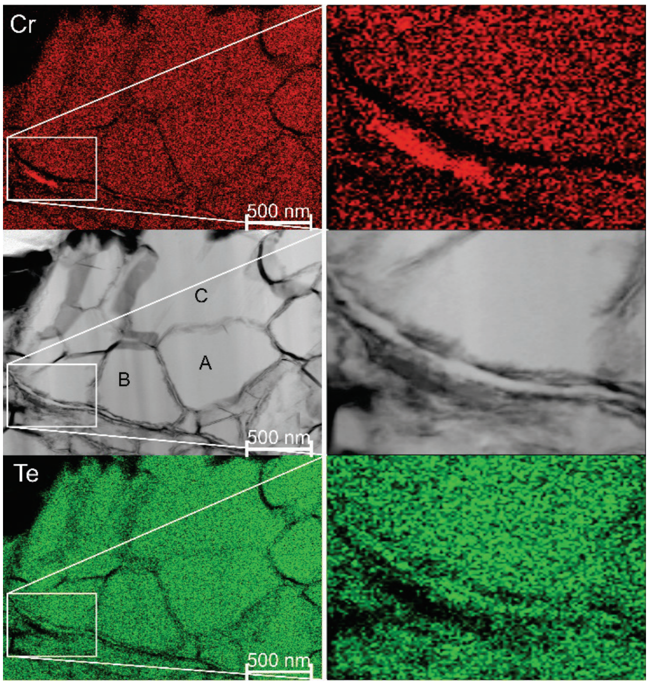

Figure S7: STEM EDX maps showing multiple grains of the quenched high-pressure phase of $CrTe_3$. The individual chemical composition of the grains denoted as A-C are summarized in Table S1 of the supporting information. The enlarged frame shows a grain boundary area containing separation into Cr- and Te-rich regions.

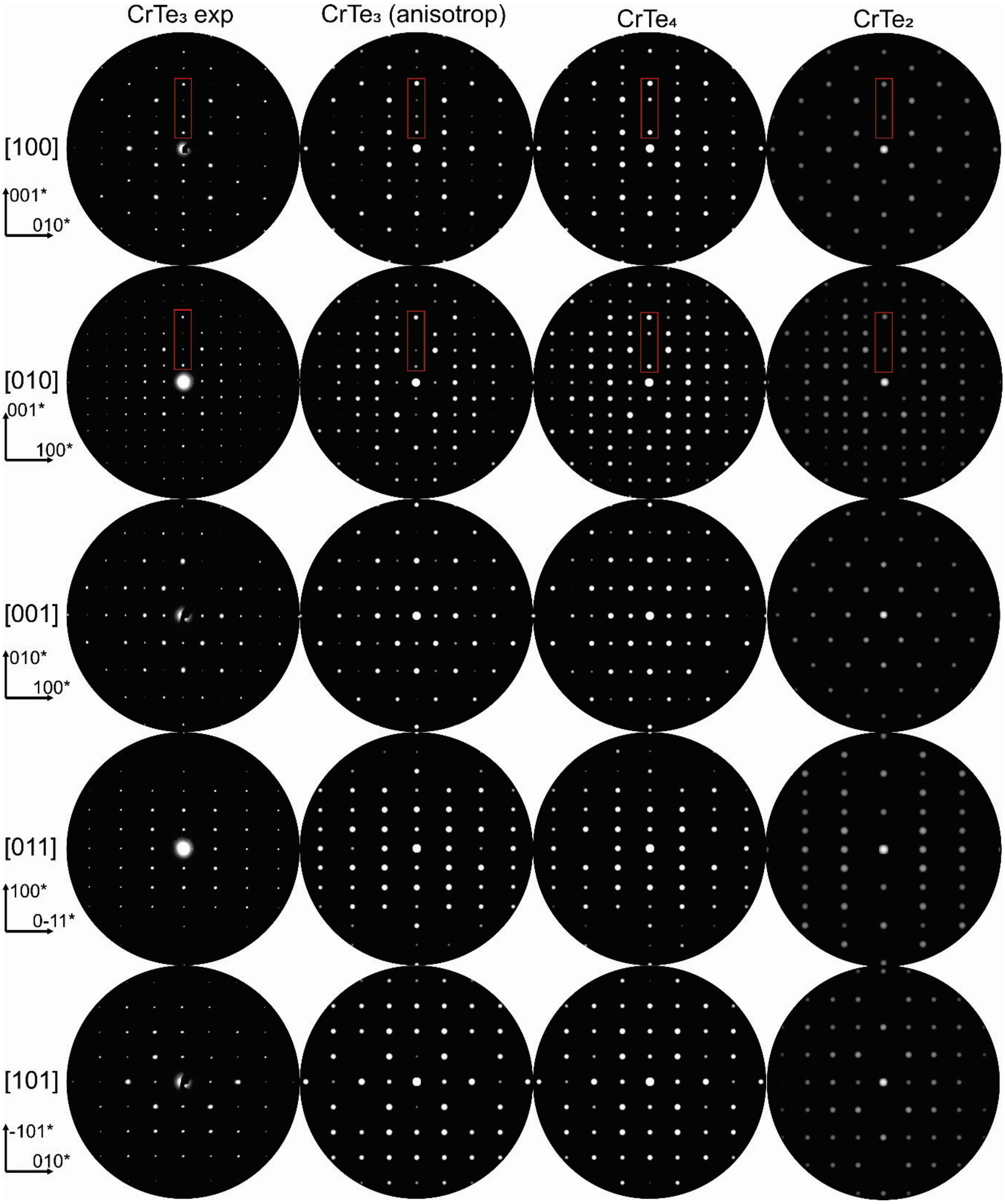

Figure S8: Recorded PED pattern and simulated patterns of the proposed models for $CrTe_2$, $CrTe_3$ and $CrTe_4$

Table S1: EDX quantification statistics of the powder measured in SEM and the EDX quantifications of the grains depicted in the cross-section STEM images micrographs of Figure 5.

EDX quantification	Cr /at.%	Te /at.%
Max (SEM)	26.03	75.29
Min (SEM)	24.71	73.97
Average (SEM)	25.52	74.48
Standard Deviation (SEM)	0.46	0.46
STEM EDX Grain A	23.20	76.80
STEM EDX Grain B	22.96	77.04
STEM EDX Grain C	23.07	76.93
STEM Average composition	23.23	76.77

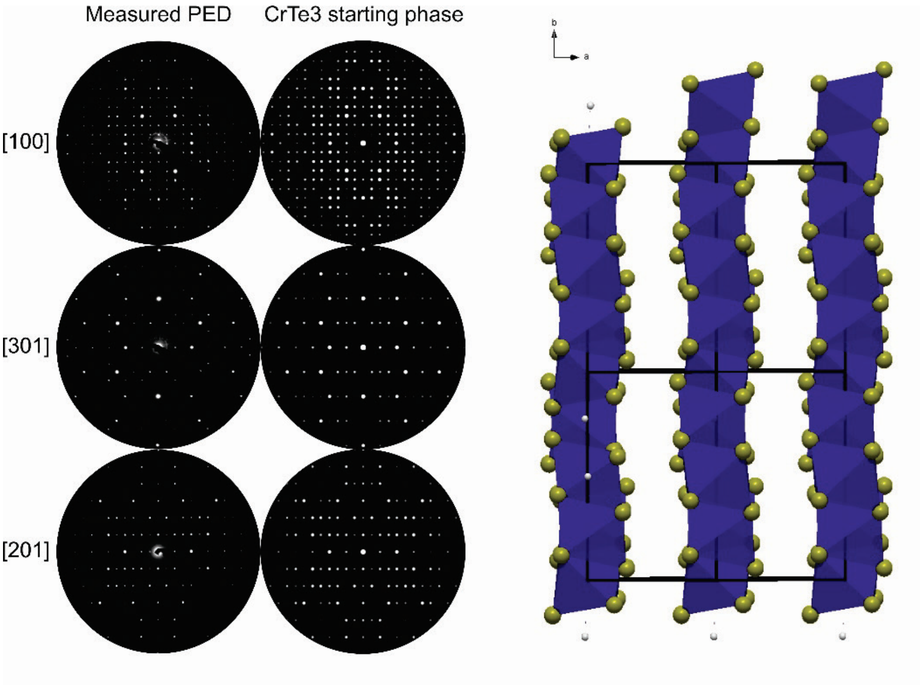

Figure S9: experimental PEDs compared to simulations of the monoclinic $CrTe_3$. The Comparison shows that the initial powder is not completely transformed into the high-pressure phase.

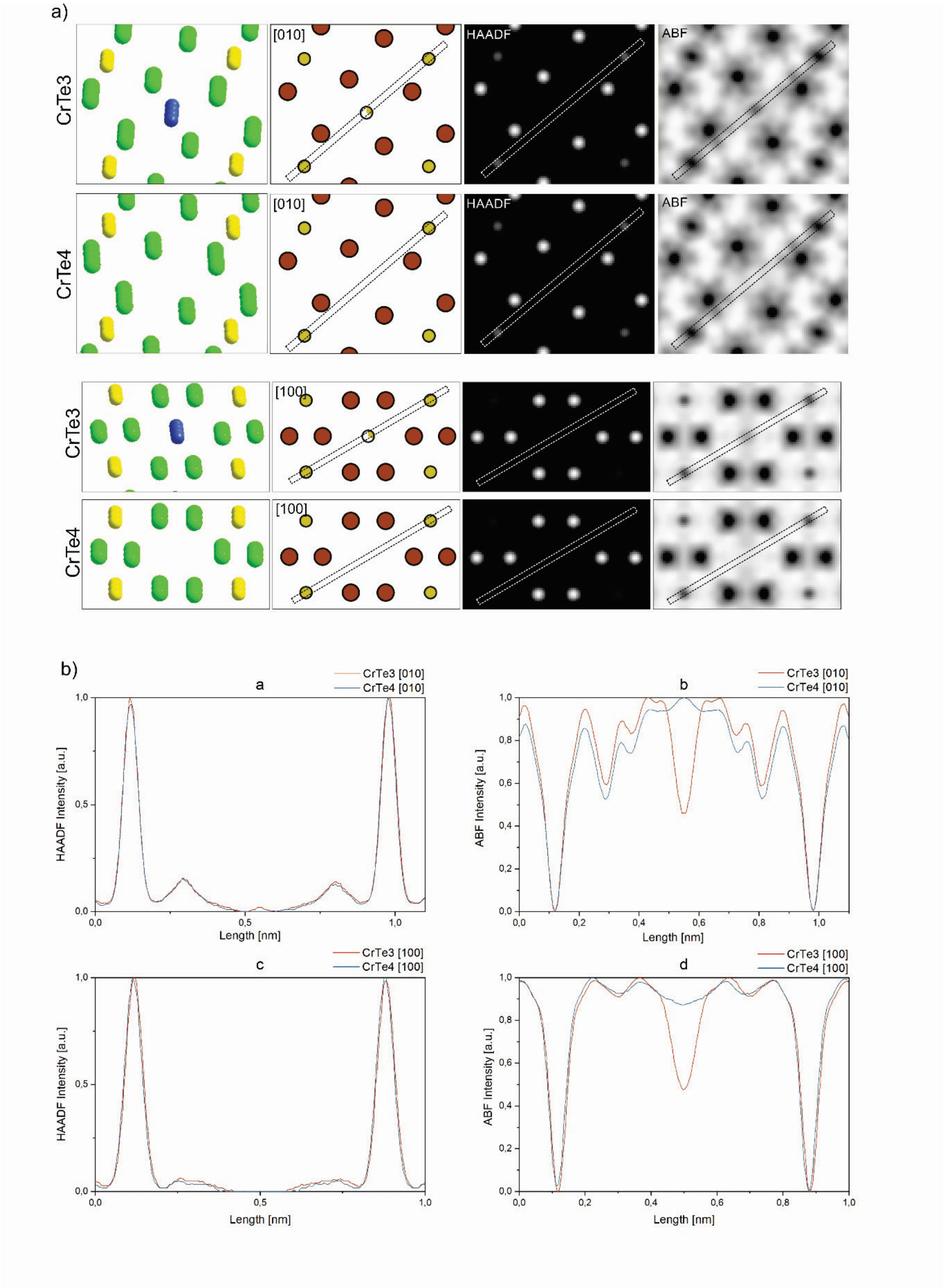

Figure S10: a) Crystal structure and regarding Dr. Probe simulation of the HAADF and ABF image in [010] and [100] direction with the view at the stacked partially occupied or empty Cr2 position in blue. In b) are the corresponding intensity profiles shown along the marked line.
